# A missense variant in *FTCD* is associated with arsenic metabolism and toxicity phenotypes in Bangladesh

**DOI:** 10.1371/journal.pgen.1007984

**Published:** 2019-03-20

**Authors:** Brandon L. Pierce, Lin Tong, Samantha Dean, Maria Argos, Farzana Jasmine, Muhammad Rakibuz-Zaman, Golam Sarwar, Md. Tariqul Islam, Hasan Shahriar, Tariqul Islam, Mahfuzar Rahman, Md. Yunus, Vincent J. Lynch, Devin Oglesbee, Joseph H. Graziano, Muhammad G. Kibriya, Mary V. Gamble, Habibul Ahsan

**Affiliations:** 1 Department of Public Health Sciences, The University of Chicago, Chicago, IL, United States of America; 2 Department of Human Genetics, The University of Chicago, Chicago, IL, United States of America; 3 Comprehensive Cancer Center, The University of Chicago, Chicago, IL United States of America; 4 Division of Epidemiology and Biostatistics, University of Illinois at Chicago, Chicago, IL, United States of America; 5 UChicago Research Bangladesh, Mohakhali, Dhaka, Bangladesh; 6 Research and Evaluation Division, BRAC, Dhaka, Bangladesh; 7 International Centre for Diarrhoeal Disease Research, Bangladesh, Dhaka, Bangladesh; 8 Department of Laboratory Medicine and Pathology, Mayo Clinic College of Medicine, Rochester, MN, United States of America; 9 Department of Environmental Health Sciences, Mailman School of Public Health, Columbia University, New York, NY, United States of America; 10 Department of Medicine, The University of Chicago, Chicago, IL, United States of America; 11 Institute for Population and Precision Health, The University of Chicago, Chicago, IL, United States of America; Stanford University School of Medicine, UNITED STATES

## Abstract

Inorganic arsenic (iAs) is a carcinogen, and exposure to iAs via food and water is a global public health problem. iAs-contaminated drinking water alone affects >100 million people worldwide, including ~50 million in Bangladesh. Once absorbed into the blood stream, most iAs is converted to mono-methylated (MMA) and then di-methylated (DMA) forms, facilitating excretion in urine. Arsenic metabolism efficiency varies among individuals, in part due to genetic variation near *AS3MT* (arsenite methyltransferase; 10q24.32). To identify additional arsenic metabolism loci, we measured protein-coding variants across the human exome for 1,660 Bangladeshi individuals participating in the Health Effects of Arsenic Longitudinal Study (HEALS). Among the 19,992 coding variants analyzed exome-wide, the minor allele (A) of rs61735836 (p.Val101Met) in exon 3 of *FTCD* (formiminotransferase cyclodeaminase) was associated with increased urinary iAs% (P = 8x10^-13^), increased MMA% (P = 2x10^-16^) and decreased DMA% (P = 6x10^-23^). Among 2,401 individuals with arsenic-induced skin lesions (an indicator of arsenic toxicity and cancer risk) and 2,472 controls, carrying the low-efficiency A allele (frequency = 7%) was associated with increased skin lesion risk (odds ratio = 1.35; P = 1x10^-5^). rs61735836 is in weak linkage disequilibrium with all nearby variants. The high-efficiency/major allele (G/Valine) is human-specific and eliminates a start codon at the first 5´-proximal Kozak sequence in *FTCD*, suggesting selection against an alternative translation start site. *FTCD* is critical for catabolism of histidine, a process that generates one-carbon units that can enter the one-carbon/folate cycle, which provides methyl groups for arsenic metabolism. In our study population, *FTCD* and *AS3MT* SNPs together explain ~10% of the variation in DMA% and support a causal effect of arsenic metabolism efficiency on arsenic toxicity (i.e., skin lesions). In summary, this work identifies a coding variant in *FTCD* associated with arsenic metabolism efficiency, providing new evidence supporting the established link between one-carbon/folate metabolism and arsenic toxicity.

## Author summary

Chronic exposure to arsenic through food and drinking water is a serious global health issue, as arsenic can increase risk for cancer, cardiorespiratory diseases, and other chronic conditions. Ingested arsenic absorbed into the blood stream is metabolized (through reduction and methylation reactions) in order to facilitate excretion in urine and removal from the body. Individuals differ with respect to the efficiency of this metabolism, in part due to inherited genetic variation. The only region of the genome known to contain variation that impacts arsenic metabolism efficiency is 10q24.32, and these variants likely alter the function of the nearby gene *AS3MT* (arsenite methyltransferase). In order to identify new genetic variants that affect arsenic metabolism, we measured variation in protein-coding regions across the entire genome for >4,800 individuals with varying levels of exposure to arsenic through naturally-contaminated drinking water in Bangladesh. Using this data, we identified a variant in the *FTCD* gene (formiminotransferase cyclodeaminase) that is associated with arsenic metabolism efficiency and risk for arsenic-induced skin lesions. This genetic variant alters the *FTCD* amino acid sequence, potentially disrupting a cryptic protein translation start site in exon 3. *FTCD* codes for an enzyme involved in histidine catabolism and one-carbon/folate metabolism; thus, our result provides new evidence supporting the well-established hypothesis that the folate/one-carbon cycle plays an important role in arsenic-related disease.

## Introduction

Exposure to inorganic arsenic (iAs) through consumption of contaminated drinking water is a major global health problem. Over 130 million individuals worldwide are exposed at levels >10 μg/L, including ~50 million in Bangladesh, where natural contamination of ground water is a well-known public health issue [1]. Arsenic is a human carcinogen [2], and chronic exposure to iAs through drinking water exceeding 50–100 μg/L is associated with various types of cancer in multiple populations [3,4] including the United States [5]. Arsenic exposure has also been linked to diabetes [6], cardiovascular disease [7], non-malignant lung disease [8], and overall mortality [9]. Arsenic-induced skin lesions are an early sign of arsenic exposure and toxicity [10] and are a risk factor for subsequent cancer [11].

Once absorbed into the blood stream, iAs can be converted to mono-methylated (MMA) and then di-methylated (DMA) forms of arsenic, with methylation facilitating the excretion of arsenic in urine [12]. This metabolism is believed to occur primarily in the liver [13]. The relative abundance of these arsenic species in urine (iAs%, MMA%, DMA%) varies across individuals and represents the efficiency with which an individual metabolizes arsenic. Arsenic metabolism is influenced by lifestyle and demographic factors [14], as well as inherited genetic variation. Prior genome-wide association (GWA) [15,16], linkage [17], and candidate gene studies [18] have shown that variation in the 10q24.32 region near the *AS3MT* gene (arsenite methyltransferase) influences arsenic metabolism efficiency, with two independent association signals observed in this region among exposed Bangladeshi individuals. These metabolism-related single nucleotide polymorphisms (SNPs) appear to impact the production of DMA (not the conversion of iAs to MMA) [14], and DMA%-increasing alleles are also associated with reduced risk for arsenic-induced skin lesions via a SNP-arsenic (i.e., gene-environment, GxE) interaction [16].

Other than 10q24.32/*AS3MT*, we currently know of no other regions of the human genome that contain variants that show robust and replicable evidence of association with arsenic metabolism efficiency [14], although studies of heritability suggest that additional variants are likely to exist [19,20]. In order to identify additional genetic variants that influence arsenic metabolism efficiency, we conducted a whole-exome study of associations between nonsynonymous, protein coding variation and arsenic metabolism efficiency.

## Results/Discussion

**Fig 1:**
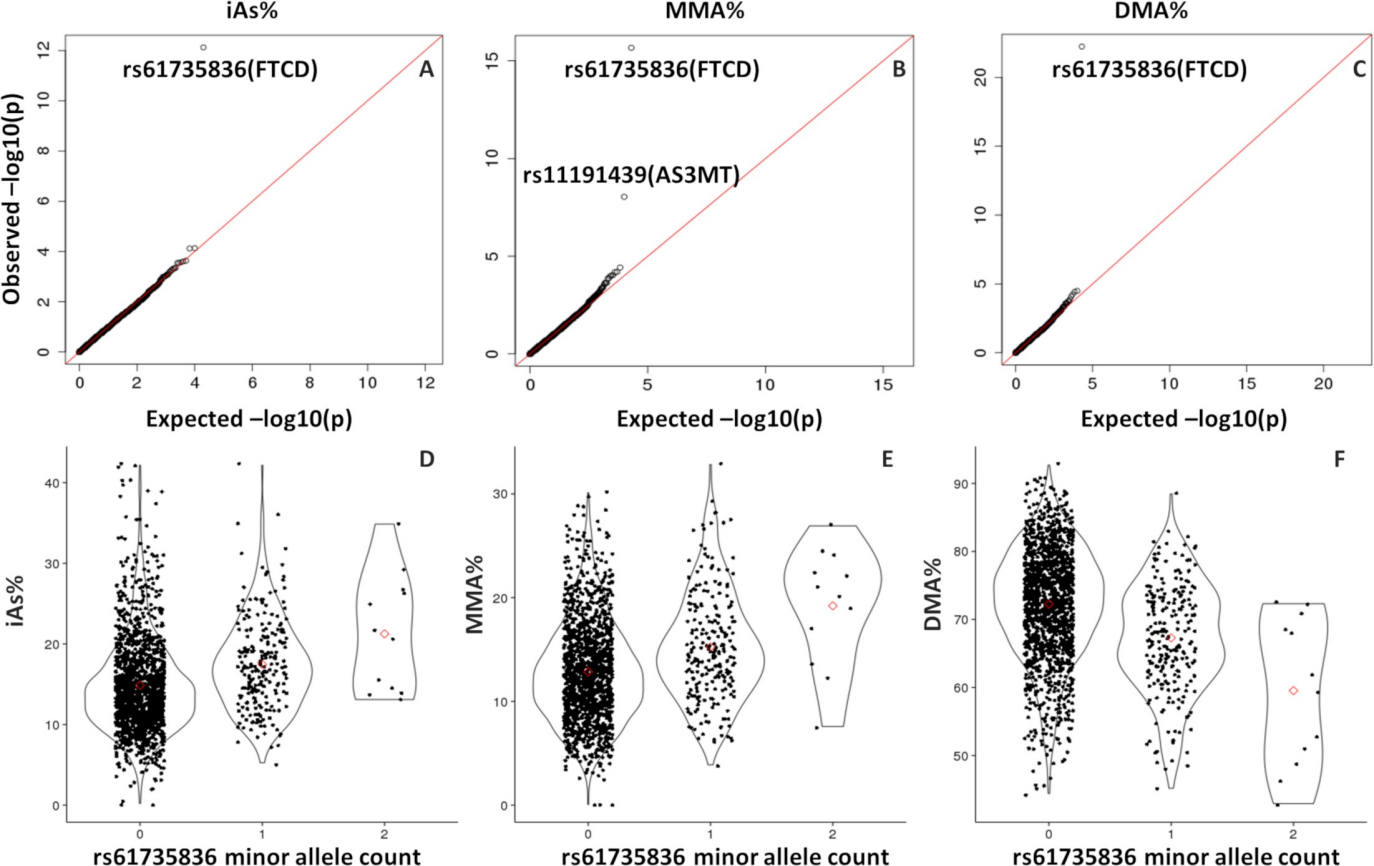
*FTCD* SNP rs61735836 is associated with the all three arsenic species measured in urine (iAs%, MMA%, and DMA%). Quantile-quantile plots (A-C) for all 19,992 post-QC exome chip variants and scatterplots (D-F) depicting the association between rs61735836 (301G>A, Val101Met) and arsenic species percentages (iAs% on left, MMA% center, and DMA% right) among 1,660 HEALS participants.

Using DNA from individuals participating in HEALS (Health Effects of Arsenic Longitudinal Study), we conducted exome-wide association analyses for each of the three major arsenic species measured in urine, using percentages of total arsenic as our primary phenotypes (iAs%, MMA%, and DMA%). For this analysis, we restricted to 1,660 genotyped HEALS participants (among 2,949 HEALS participants with Illumina exome array data) with available data on arsenic species in urine. After SNP QC (see methods), we had data on 19,992 variants with MAF >1%, and ~90% of these were missense variants. Among these SNPs, rs61735836 (chr21:47572637 based on hg19) showed a clear association with all three arsenic species percentages (**Fig 1A–1C**). P-values for this association were P = 8x10^-13^ for iAs%, P = 2x10^-16^ for MMA%, and P = 6x10^-23^ for DMA%. The minor allele (A) was associated with decreased DMA% and increased MMA% and iAs% (**Fig 1D–1F**), consistent with the directions of association previously observed for SNPs in the *AS3MT* region. Results for all 19,992 variants are in **Supporting Files S1-S3**.

Like *AS3MT*, this association was most relevant to the second methylation step, as it showed a strong association with the secondary methylation index (SMI = DMA/MMA), but not the primary methylation index (PMI = MMA/iAs) (**S1 Table**). Similarly, after applying principal components (PC) analysis to arsenic species percentages as previously described [14], rs61735836 showed strong association with PC1 (representing production of DMA) but not PC2 (representing conversion of iAs to MMA) (**S1 Table**). Individuals carrying two minor alleles (AA) as compared to one (AC) appear to have even lower DMA%, suggesting a potential additive effect of the A allele; however, our sample size of minor allele homozygotes was small (n = 12), limiting our ability to examine differences between these two groups (**S1 Table**). The association of rs61735836 with arsenic species was similar across groups stratified by sex and age (**S2 Table**), and rs61735836 did not show evidence of interaction with either of the AS3MT SNPs previously identified in this population (rs9527 and rs11191527) in relation to DMA% or skin lesions status (**S3 Table**). The probe intensity data for rs61735836 is shown in **S1 Fig**, with very distinct clusters indicating high-quality data for this SNP.

We then conducted exome-wide association analyses of arsenical skin lesion status (the most common sign of arsenic toxicity) using data on 2,401 cases and 2,472 lesion-free controls (from both HEALS and BEST, the Bangladesh Vitamin E and Selenium Trial). While there was no notable departure from the expected null distribution, the low-efficiency allele for *FTCD* SNP rs61735836 (A) was associated with increased skin lesion risk (per allele OR = 1.25; P = 5x10^-4^; risk allele carrier OR = 1.35, P = 1x10^-5^) (**S2 Fig**). Results for all 19,992 variants are in **S4 File**. This observation is similar to what has been observed for metabolism-related variants in the *AS3MT* region and suggests rs61735836 impacts arsenic toxicity risk through its impact on arsenic metabolism efficiency. In this manner, this variant would be expected to reduce urinary arsenic elimination and thereby increase the internal or biologically effective dose of arsenic.

The MAF for rs61735836 was 0.077 in our data, highly consistent with the MAFs of 0.064 and 0.079 observed in the 1,000 Genomes Project (1KG) Bangladesh (BEB) population and South Asian (SAS) super-population, respectively. The MAF for this variant is less than <21% in all human populations with available data in the Geography of Genetic Variants browser [21] and is most common in East Asian populations (**S3 Fig**).

**Fig 2:**
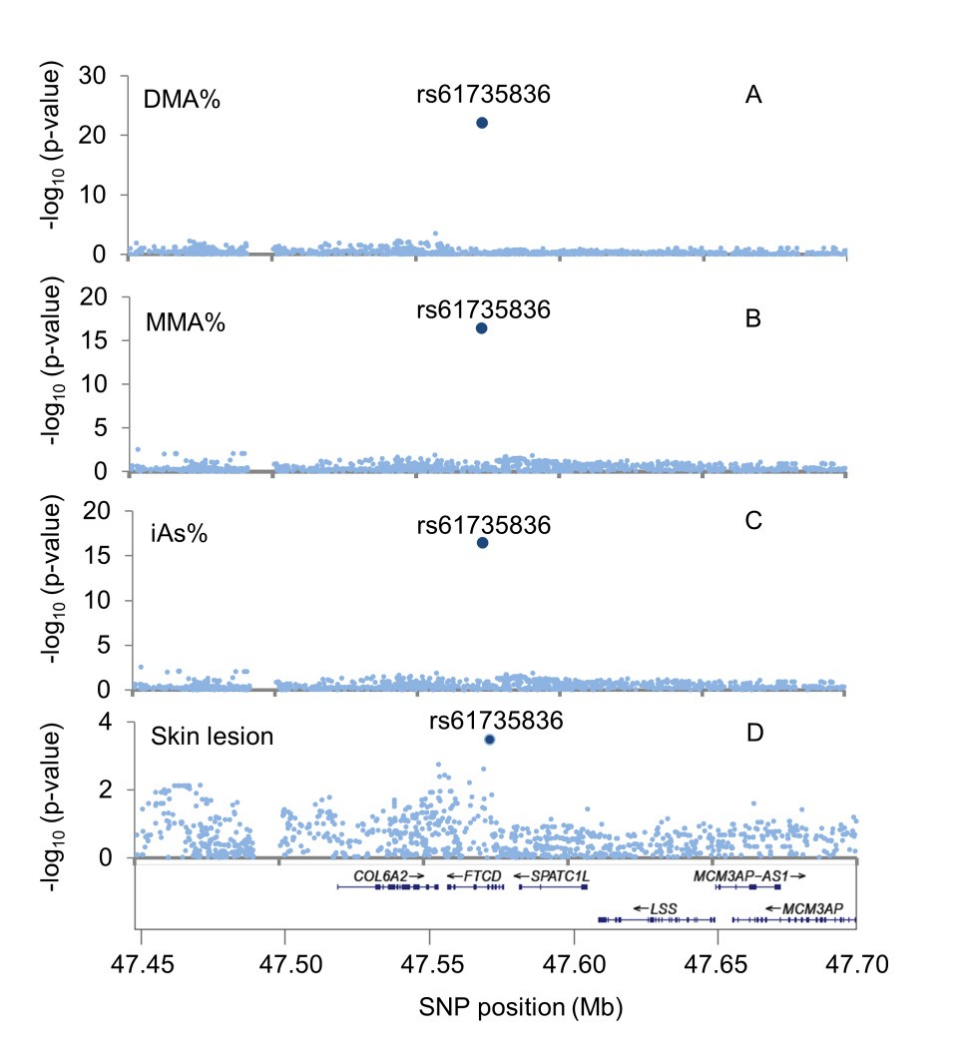
Regional association plots for the *FTCD* region (21q22.3). The vertical axes show the–log_10_(P-value) for the association of SNP allele counts with (A) DMA%, (B) MMA%, (C) iAs% (A-C based on 1,660 HEALs participants), and (D) arsenic induced skin lesions (2,401 cases, 2,472 controls).

After combining our exome array results with our previously reported GWA results for genome-wide SNPs [15,16] (HumanCytoSNP-12 array imputed to ~8.2 million SNPs using 1KG phase 3 v5), we observed that rs61735836 is the only variant in this region showing strong evidence of association (**Fig 2**). This is consistent with the observation that rs61735836 is not in linkage disequilibrium (LD) (r^2^>0.1) with any nearby variant in 1KG South Asian (SAS) populations. This SNP is in mild LD with nearby variants in the 1KG African (AFR) super-population (r^2^~0.27) (**S4 Fig**), with the strongest LD observed in the ESN (Esan in Nigeria) population (r^2^ = 0.43 with rs184976755). SNP rs61735836 was not genotyped in our prior GWA study [15,16], and therefore could not be imputed due to the lack of LD with nearby variants. Among the 5 additional exonic variants in *FTCD* that passed QC (all missense), none showed association with any of our arsenic species measures (P>0.01).

Using data from HEALS, we tested rs61735836 for evidence of interaction with baseline arsenic exposure in relation to risk for arsenic-induced skin lesions (which were primarily incident lesions diagnosed after baseline). As an exposure measure, we used the arsenic concentration measured in the drinking well that each individual reported as their primary water source at baseline (prior to arsenic mitigation efforts in the HEALS cohort [22]). A test of multiplicative interaction produced a non-significant sub-multiplicative interaction estimate (OR = 0.86, P = 0.42), while a test of additive interaction produced a non-significant supra-multiplicative interaction (RERI = 0.49; P = 0.10) (**Table 1**).

**Table 1:** Odds ratios (ORs) for the association between rs61735836 carrier status and arsenic-induced skin lesions, including exposure-stratified ORs.

	GG		GA or AA	
	Case/control	OR (CI)	Case/control	OR (CI)
**All subjects**	1,955/2,137	1.00 (Ref)	446/355	1.35 (1.18,1.54)
	P-value = 1x10^-5^			
**By Exposure Tertile**^**1**^	363/2,142		80 /329	
**1**	64/786	1.00 (ref.)	14/97	1.66 (0.85,3.22)
**2**	125/690	2.31 (1.64,3.26)	29/115	3.75 (2.19,6.41)
**3**	174/640	4.13 (2.96,5.77)	80/110	5.03 (3.08,8.32)
	Multiplicative interaction OR = 0.86 (0.59, 1.25); P = 0.42			
	Additive Interaction RERI = 0.49 (-0.09, 1.08); P = 0.10			

RERI, relative excess risk due to interaction. All models are adjusted for age, sex, BMI, smoking, and socioeconomic variables (education, land ownership, and TV ownership).

^**1**^ Only HEALS participants were used for exposure-stratified and SNP-arsenic interaction analyses. BEST participants were not included to due lack of an exposure measurement taken prior to arsenic mitigation efforts.

To further assess the impact of rs61735836 on arsenic metabolism, we obtained data on arsenic species in blood (as opposed to urine) for 155 of our genotyped HEALS cohort members. These HEALS participants had existing data on arsenic metabolites in blood due to their participation in additional arsenic-related studies focused on folic acid and/or creatinine supplementation [23,24] and oxidative stress [25]. Consistent with our observed association with arsenic species in urine, the minor allele of rs61735836 (A) showed evidence of association with decreased DMA% (P = 0.02), increased MMA% (P = 0.41), and increased iAs% (P = 0.02), with arsenic species measured prior to any intervention (**S4 Table**). Among these 155 participants, 109 also had data on arsenic species in blood collected 12 weeks after the start of a supplementation intervention. Under the assumption that the interventions do not modify the impact of rs61735836 on arsenic metabolism efficiency (an assumption we make with considerable uncertainty), we can also examine these associations using these post-intervention measures. Using a mixed-effects model to analyze data from both time points, we observed that the A allele is associated with decreased DMA% (P = 0.005), increased MMA% (P = 0.01), and increased iAs% (P = 0.15) (**S4 Table**), consistent with results based on arsenic species measured in urine.

**Fig 3:**
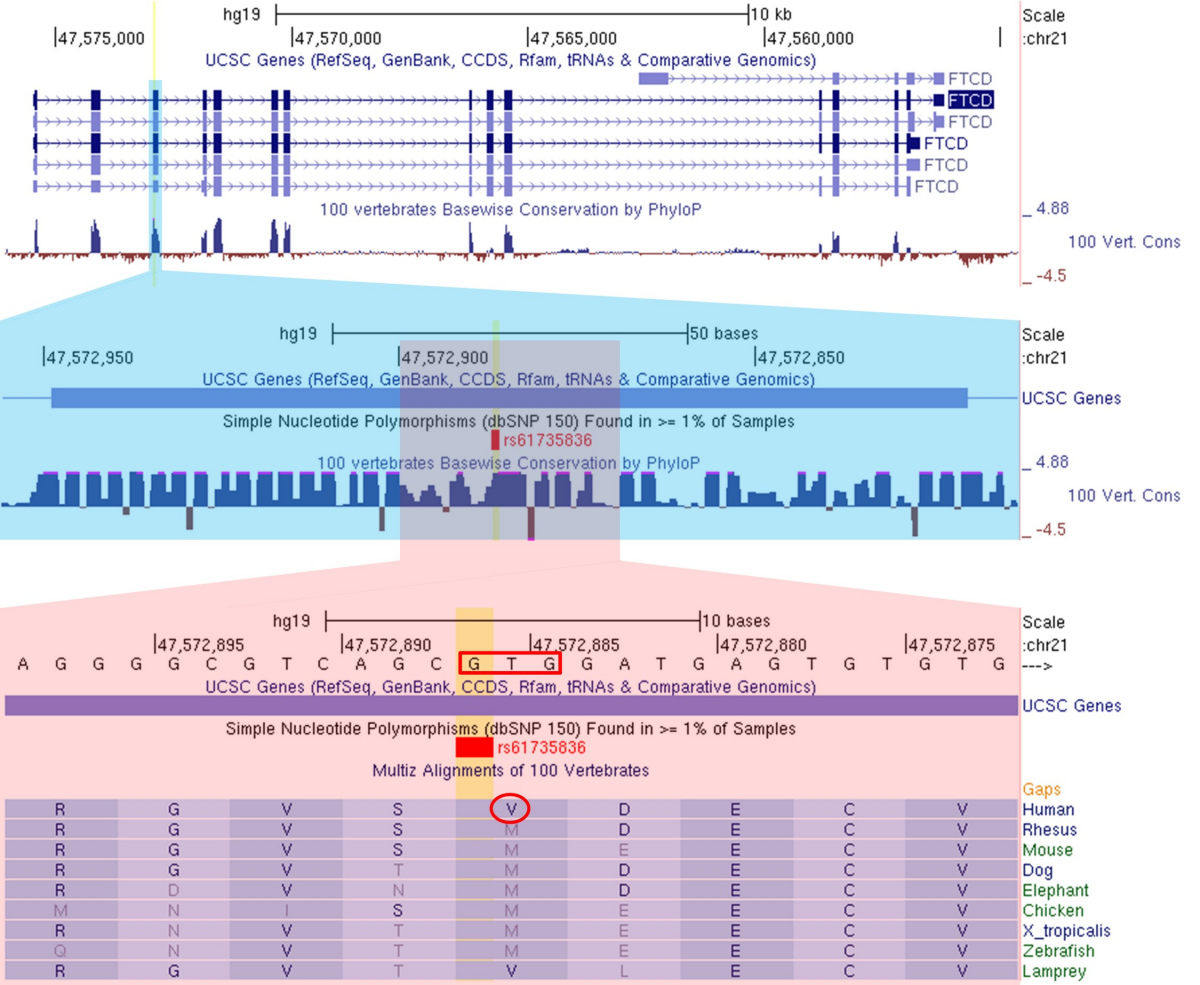
The minor allele of missense variant rs61735836 changes a valine to a methionine. The minor allele (A, MAF = 0.07) changes a valine (V, circled) codon (GTG, red box) to a methionine (M) codon (ATG/AUG) at a site that is highly conserved across vertebrates. This change introduces a potential start codon in exon 3 which is the first 5´-proximal Kozak consensus sequence ([A/G]xxAUGG) in the *FTCD* gene.

SNP rs61735836 resides in exon 3 of *FTCD* (Formiminotransferase cyclodeaminase), a gene predominantly expressed in liver [26,27] (**S5 Fig**), the tissue in which the majority of arsenic metabolism is believed to occur [13]. *FTCD* codes for a 541-amino-acid protein that forms a homo-octameric enzyme involved in histidine catabolism. SNP rs61735836 codes for a valine to methionine substitution at codon 101 (p.Val101Met) (**Fig 3**). The major (G) and minor (A) alleles correspond to valine and methionine, respectively. Codon 101 codes for an amino acid in the formiminotransferase N-subdomain and resides between secondary structure elements β4 and α4. This codon is highly conserved [27] with methionine being the predominant amino acid in all other vertebrates, including the Neanderthal and Denisovan sequences (with the exception of lamprey, which is Valine) (**Fig 3**). This suggests the derived Valine codon (G allele) has gone to near fixation in humans at some point after the modern-archaic human split, suggesting selection on a functional mutation (G) that confers a selective advantage.

We do not yet understand the mechanism by which rs61735836 presumably affects arsenic metabolism; however, there are several mechanisms by which rs61735836 may affect *FTCD* function. First, because the minor/ancestral allele A produces a start codon (Met), this allele may create an alternative translation start site that would produce a truncated *FTCD* protein. The minor allele A/Met creates the first 5´-proximal Kozak consensus sequence in the *FTCD* gene ([A/G]xxAUGG). While translation generally initiates at the first 5´ AUG, the efficiency with which this AUG is recognized is influenced by the presence of a Kozak consensus sequence [28]. For 5´-proximal AUG codons that do not reside in a Kozak consensus sequence, ribosomes can fail to initiate translation at that site, and continue scanning for downstream start codons (i.e., “leaking scanning”) [29]. There are three start codons upstream of rs61735836, but none are a Kozak consensus sequence, including the canonical start site (**S6 Fig**).

Second, the V → M amino acid substitution may alter the structure of the protein, potentially through protein folding or octamer formation, thereby altering the efficiency with which the *FTCD* enzyme functions. However, this substitution is not strongly predicted to be damaging according to SIFT (“tolerated” with a score of 1.0), PolyPhen-2 (benign with a score of 0.029), CADD (0.77 with a PHRED-like scaled score of 9.3), and ClinVar (likely benign).

Third, exon 3 is just downstream of several transcription factor binding sites and chromatin marks indicative of enhancers and promoters, and the exon itself is contained within a weak promoter in the HepG2 liver cancer cell line (**S7 Fig**). This suggests that it is possible that rs61735836 could affect initiation of transcription or represent a translation start site specific to an *FTCD* isoform that lacks the canonical start codon. However, among the 14 *FTCD* isoforms observed in GTEx liver tissue, no transcripts lacking exon 1 include exon 3 (**S8 Fig**). Furthermore, rs61735836 is not associated with *FTCD* expression in any GTEx tissue, including liver, and is not reported to be an *FTCD* isoform QTL, suggesting that the effect of this SNP is likely due to the amino acid substitution.

**Fig 4:**
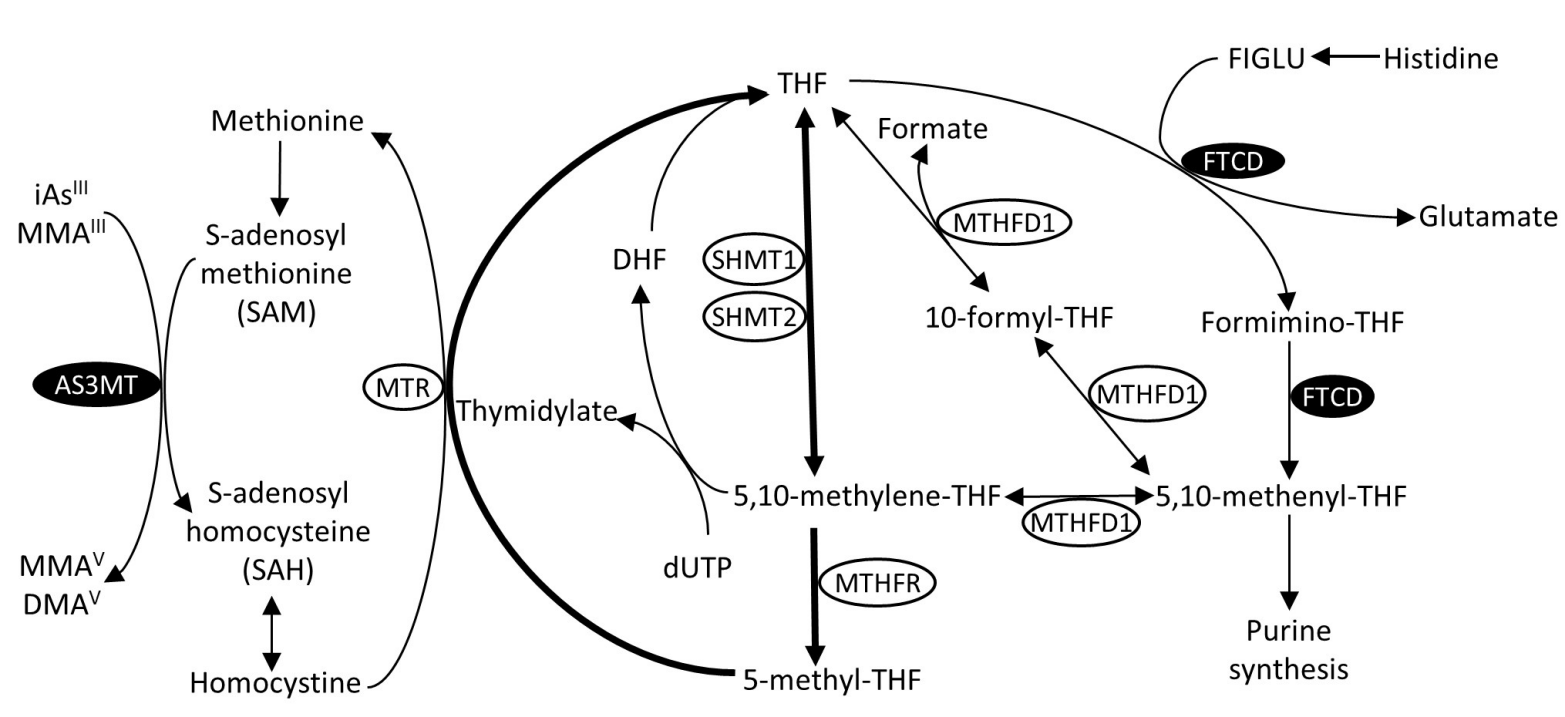
The role of *FTCD* in histidine catabolism and the one-carbon/folate cycle, which provides methyl groups for arsenic methylation (by *AS3MT*) via the methionine cycle. The formininotranserase domain of *FTCD* catalyzes the transfer of a formimino group from N-formimino-L-glutamate (FIGLU) (or a formyl group from N-formyl-L-glutamate) to tetrahydrofolate (THF) producing formimino-THF. The cyclodeaminase domain of *FTCD* then catalyzes the removal of ammonia from formimino-THF, generating 5,10-methenyl THF, which can then be converted to 5:10 methylene-THF or THF, both key components of the canonical one-carbon/folate cycle (shown in bold). The folate cycle contributes one-carbon groups to the methionine cycle, which in turn supplies these groups to methyltransferases (such as *AS3MT*) involved in methylation of arsenic, DNA, and other substrates. DHF, dihydrofolate; dUTP, deoxyuridine triphosphate.

The enzyme encoded by *FTCD* catalyzes the two consecutive final reactions of the L-histidine degradation pathway, which links histidine catabolism to one-carbon/folate metabolism (**Fig 4**) [27]. First, the formiminotranserase domain of *FTCD* catalyzes the transfer of a formimino group from N-formiminoglutamate (FIGLU) to tetrahydrofolate (THF), freeing glutamate and adding a one-carbon substituent at the oxidation level of formic acid to THF. Second, the cyclodeaminase domain catalyzes the removal of ammonia from formimino-THF, generating 5,10-methenylTHF [30,31]. *MTHFD1* catalyzes the interconversion of 5,10-methenylTHF to either 5,10-methyleneTHF or to THF (via 10-formylTHF), both of which can enter the folate cycle and be used for synthesis of 5-methylTHF. Histidine has been proposed as a potential source 5,10-methenyl-THF in some tissues [32]; however, the relative contribution of histidine to the one-carbon pool is currently unclear, and contribution may vary across tissues [33]. Additional potential roles of *FTCD* include catalyzing the conversion of THF to 5-formyl-THF and conversion of 5-formyl-THF to 5,10-methenyl-THF [34,35].

The one-carbon cycle is critical for arsenic metabolism, because 5-methyl-THF (primarily originating from dietary sources, but also generated from histidine catabolism) is essential to the production of S-adenosylmethionine (SAM) which provides methyl groups for methyltransferase reactions, including methylation of arsenic (**Fig 4**). Methylation of arsenic is catalyzed by *AS3MT*, a known arsenic susceptibility/metabolism gene [15,18]. The methionine cycle is also linked to the production of glutathione (GSH), which may increase the speed of arsenic reduction (i.e., arsenate (As^V^) to arsenite (As^III^)), which occurs prior to methylation of arsenic by *AS3MT*. Variation in folate status/intake and one-carbon metabolism have long been hypothesized to influence arsenic metabolism [36], and randomized studies have provided strong evidence that folate supplementation increases arsenic metabolism efficiency and reduces blood arsenic concentrations [23,37]. However, prior candidate gene association studies of polymorphisms in one-carbon metabolism genes and arsenic metabolism have provided only suggestive or null findings [38,39], and no prior studies examined variation in *FTCD*.

Interestingly, a recent GWA study of 124 arsenic-exposed women living in the northern Argentinean Andes identified associations between SNPs in the 21q22.3 region and urinary DMA% (P = 1.2x10^-5^) and MMA% (P = 1.2x10^-5^) (Schlebusch et al [40]). The SNPs showing the strongest associations reside in the LSS, MCM3AP, and YBEY genes, which are in the range of ~30 to ~150 kb upstream of (and telomeric to) FTCD. While this previously reported signal is nearby the signal we report, the two signals appear distinct. Our association involves a single coding SNP in FTCD that is in very low LD with all surrounding SNPs, while the Schlebusch et al. association involves many SNPs in a LD block that spans several genes (with no association observed for SNPs within FTCD itself). Thus, it appears unlikely these two signals are due to the same causal variant. However, it is possible that the causal variants underlying these associations impact the function of the same gene(s).

As of January 31, 2019, the *FTCD* gene has not been reported in any GWA study of human traits (according to the NHGRI-EBI GWAS catalog). Due to the very weak LD between rs61735836 and nearby variants, this variant cannot be accurately imputed in most populations; it must be directly genotyped. However, commercially available arrays that lack “exome content” (https://genome.sph.umich.edu/wiki/Exome_Chip_Design) do not include rs61735836. Among arrays used in prior GWA studies, 25 (out of 56) Illumina arrays and 1 (out of 20) Affymetrix array include rs61735836 (based on LDlink [41]). Thus, a large fraction of prior GWA studies have not measured or imputed rs61735836, including all studies conducted prior to the development of the exome content.

Rare mutations in *FTCD* cause various forms of *FTCD* deficiency (OMIM: 229100), an autosomal recessive disorder which is the second most common inborn error of folate metabolism [31,42]. Severe forms have been reported to cause mental and physical retardation, anemia, and elevated serum folate, while less severe cases have been reported to have developmental delay and elevated levels of FIGLU in urine [30], which accumulates due to FTCD deficiency (**Fig 4**). Recent work has demonstrated that individuals homozygous for putative loss-of-function mutations in FTCD have clearly detectable levels of FIGLU in urine in the absence of histidine loading (which is normally very low or undetectable), in the range of 5 to 195 mmol per mol creatinine [43].

To assess the potential impact of rs61735836 on urine FIGLU, we measured FIGLU in baseline urine samples for 60 of our HEALS participants (20 for each of the three rs61735836 genotype categories) using tandem mass spectrometry in the laboratory of Dr. Devin Oglesbee as described previously [43]. We observed no evidence for elevated FIGLU among carriers or non-carriers of the G allele, with no participant having a FIGLU >0.25 mmol/mol creatinine (**S9 Fig**). This finding suggests that impact of rs61735836 on FTCD function is less severe than the impact of loss of function mutations on FIGLU.

**Fig 5:**
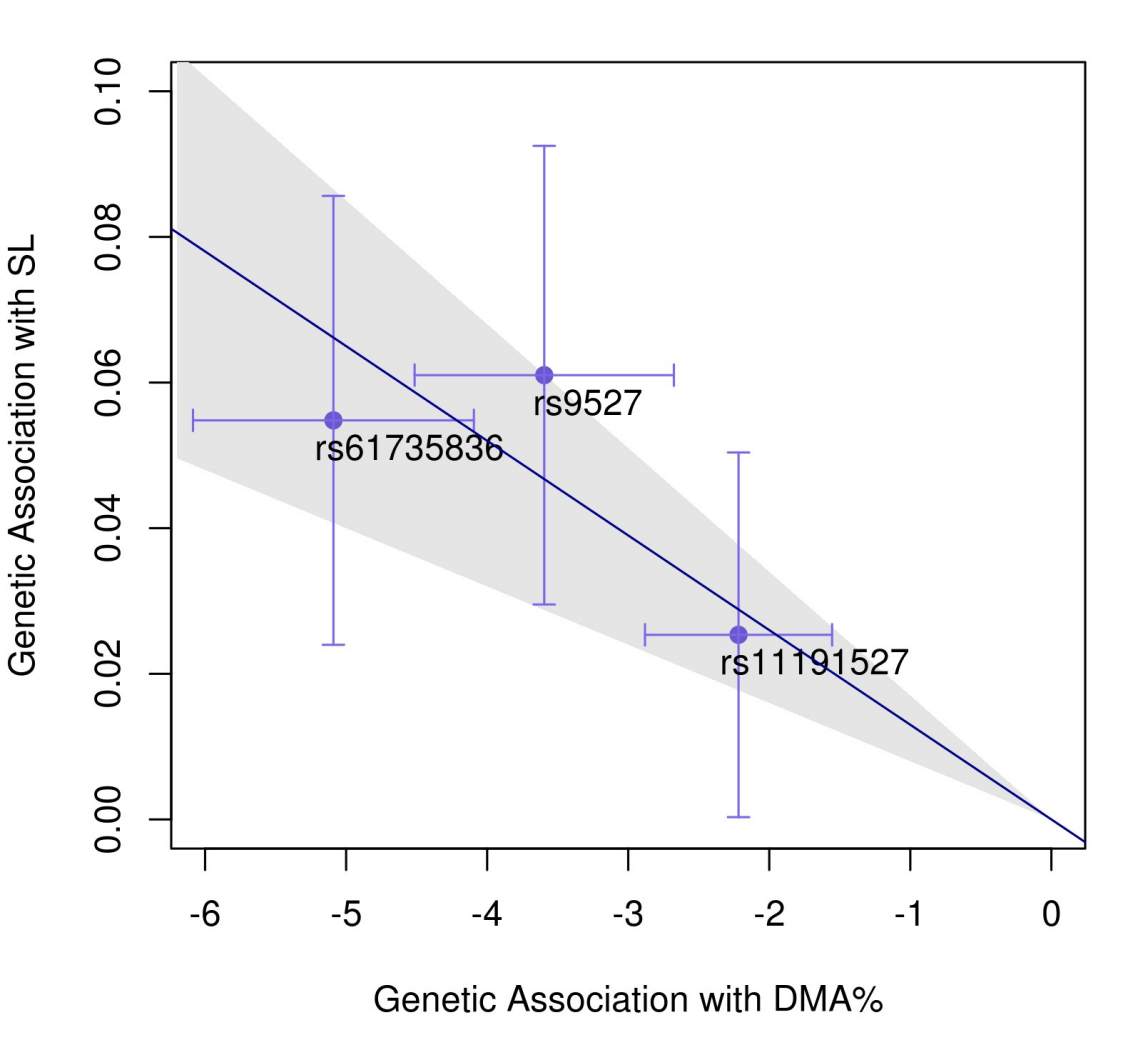
Mendelian randomization supports a causal effect of arsenic metabolism efficiency on arsenic-induced skin lesion risk. Horizontal and vertical error bars for each SNP correspond to the 95% CI for the beta coefficient for its association with DMA% and skin lesion risk, respectively. The slope of the diagonal line (-0.013) is the inverse-variance-weighted estimate of the causal effect (i.e., the ln(OR), corresponding to OR = 0.89 for a 10% increase in DMA%; P = 6x10^-8^).

Combining data on *FTCD* SNP rs61735836 with the two previously-reported arsenic metabolism SNPs in the *AS3MT* region (rs9527 and rs11191527) [15,16], we can explain ~10% of the phenotypic variation in DMA% for our HEALS participants. Mendelian randomization analyses of all three variants (using the inverse-variance weighted meta-analysis method [44]) provides strong evidence of a causal effect of arsenic metabolism efficiency (as measured by DMA%) on skin lesion (OR = 0.89 for a 10% increase in DMA%; P = 6x10^-8^) (**Fig 5**). We observe similar results when using (a) either iAs% or MMA% as a measure metabolism efficiency and (b) alternative MR methods implemented in the MendelianRandomization R package [44] (**S10 Fig**).

These MR results are consistent with prior observational studies [14,45–48] showing that high DMA% (and SMI) are generally associated with decreased skin lesion risk, while high iAs%, MMA%, and PMI are generally associated with increased skin lesion risk. These observational studies also indicate that, among the various arsenic metabolism measures, MMA% is most consistently associated with increased risk for skin lesions and several types of cancer [49]. Consistently, *in vitro* studies indicate MMA^III^ is likely to be the most toxic of all metabolites of inorganic arsenic [50,51]. Thus, the primary finding from this work and our prior studies—that producing DMA more efficiently (and therefore depleting iAs and MMA) reduces skin lesion risk—could be attributed to a) enhanced excretion of arsenic from the body in the form of DMA and/or b) lower percentages of the most toxic metabolites (e.g. MMA^III^) among all arsenic species in the body.

*FTCD* SNP rs61735836 showed suggestive evidence of additive GxE interaction, results that are directionally consistent with previously reported additive interaction results for *AS3MT* genotypes [16]. For both loci, the expected interaction between SNP and arsenic exposure in relation to skin lesions is much more apparent on the additive scale compared to the multiplicative scale. This is an important observation considering these SNPs must modify the effect arsenic on skin lesion risk, a conclusion we draw based on the fact that these lesions do not occur in the absence of arsenic exposure. In other words, this variant cannot affect skin lesion risk among unexposed individuals, so GxE must be present. However, because we have few truly unexposed individuals in our study, we are unable to assess GxE on the present vs. absent exposure scale. In addition, it is possible that we are not well-powered to detect GxE due to the low MAF of rs61735836 and the relatively small number of genotyped cases having exposure data obtained prior to arsenic mitigation efforts (n = 443).

In summary, this work identifies a protein-altering variant in *FTCD* (rs61735836) that is associated with both arsenic metabolism efficiency and risk for arsenic-induced skin lesions, the most common sign of arsenic toxicity. Future studies can use this variant, in conjunction with *AS3MT* variants, to study the effects of arsenic exposure (through food, water, or other sources) and metabolism efficiency on health outcomes believed to be affected by arsenic (e.g., cancer and cardiovascular disease), even in the absence of data on arsenic exposure. This work provides evidence of links among histidine catabolism, one-carbon/folate metabolism, and arsenic metabolism, which is intriguing in light of the strong prior evidence supporting a role for folate status and one-carbon metabolism in arsenic metabolism efficiency [36], including randomized studies of folate supplementation in humans [23,37]. However, additional research is needed to understand (1) if and how this SNP impacts the relative distribution of folate metabolites and (2) the potential mediating role of folate on the association between rs61735836 and arsenic metabolism efficiency. A better understanding of these effects could enable the use of rs61735836 as a tool for studying the many human diseases with hypothesized connections to folate and one-carbon metabolism (e.g., cancer, vascular disease, cognitive decline, neural tube defects) [52–54].

## Materials and methods

### Ethics statement

This research was approved by the Institutional Review Board of the University of Chicago (IRB16-1236). Verbal informed consent was obtained from all participants.

### Participants

The DNA samples used in this work were obtained at baseline interview from individuals participating in one of the two following studies: the Health Effects of Arsenic Longitudinal Study (HEALS) [55] and the Bangladesh Vitamin E and Selenium Trial (BEST) [56]. HEALS is a prospective study of health outcomes associated with arsenic exposure through drinking water in a cohort of adults in Araihazar, Bangladesh, a rural area east of the capital city, Dhaka. A cohort of ~12,000 participants was recruited in 2000–2002, and ~8,000 additional participants were recruited in 2006–2008. Over 6,000 wells in the study area have been tested for arsenic using graphite furnace atomic absorption spectrometry and individuals reported the primary well from which they drank. Trained study physicians conducted in-person interviews, clinical evaluations (including ascertainment of skin lesions), and spot urine collection at baseline and follow-up visits (every two years). BEST is a 2×2 factorial randomized chemoprevention trial (n = 7000) evaluating the effects of vitamin E and selenium supplementation on non-melanoma skin cancer (NMSC) risk. BEST participants are residents of Araihazar (the same geographic area as HEALS), Matlab, and surrounding areas. BEST uses many of the same study protocols as HEALS, including arsenic exposure assessment and biospecimen collection. All BEST participants had existing arsenic-related skin lesions at baseline.

The exome-wide association study of arsenic species percentages was conducted using urinary arsenic metabolite and exome chip SNP data on 1,660 individuals randomly selected from HEALS. Exome-wide association analyses of arsenic-induced skin lesions were conducted using exome chip SNP data on 2,401 cases and 2,472 lesion-free controls (from both HEALS and BEST). This case-control sample includes 1,660 HEALS participants with arsenic metabolite data. Analyses of blood arsenic metabolites were conducted using 155 cohort members for whom we had existing data on arsenic species measured in blood. These data on blood arsenic species were generated in the context of various HEALS ancillary studies: the Nutritional Influences on Arsenic Toxicity (NIAT) Study [23], the Folate and Oxidative Stress (FOX) Study [25], and the Folic Acid and Creatinine Trial (FACT) [24] (data courtesy of Gamble, MV and Graziano, JH). Among these 155 participants, 147 were included in the case-control analysis of skin lesions, and 87 were included in the analysis of arsenic metabolites in urine.

We assessed SNP-arsenic interaction using data on HEALS participants with individually-measured arsenic exposure (i.e., arsenic concentration of their primary drinking well at baseline). These exposure measures were taken prior to arsenic mitigation efforts [22]; thus, these measures represent longer-term, historical exposure levels. The majority of the HEALS participants (~95%) were lesion-free at baseline. Similarly, among our genotyped HEALS participants, only 66 of the 443 skin lesion cases were prevalent cases. The remaining 377 were incident skin lesions cases (ascertained at biennial follow-up visits by trained study physicians using a structured protocol [55]). All BEST participants had skin lesions at baseline, because a skin lesion diagnosis was part of the BEST eligibility criteria [56]. In this study, skin lesion cases were defined as individuals with any type of arsenic-induced lesion, including keratosis, melanosis, and/or leukomelanosis.

Study protocols were approved by the Institutional Review Boards of The University of Chicago and Columbia University, the Ethical Review Committee of the International Center for Diarrheal Disease Research, Bangladesh, and the Bangladesh Medical Research Council. Informed consent was obtained from all participants.

### Genotyping and quality control

Using DNA from individuals participating in HEALS (Health Effects of Arsenic Longitudinal Study) and BEST (the Bangladesh Vitamin E and Selenium Trial), we genotyped 4,939 Bangladeshi individuals (HEALS n = 2,949; BEST n = 1,983) using Illumina’s exome array v1.1. Prior to QC, our dataset consisted of 242,901 variants. We removed samples with >3% missing SNPs (n = 6), gender mismatches (n = 22), and duplicate individuals (n = 25). We removed SNPs with call rate <97% (176 SNPs), monomorphic SNPs (n = 27,687), and 166 SNPs deviating from Hardy-Weinberg Equilibrium (P<10^−10^). None of the SNPs that pass this HWE threshold show HWE P-values <10^−7^ when relative pairs are removed from the dataset. We removed SNPs with a minor allele frequency (MAF) <1% (n = 178,015). Among the 19,992 post-QC variants, there were 17,919 missense, 141 nonsense, 1,260 synonymous, and 672 non-exonic variants. All post-QC variants were included in our analysis. A similar QC procedure for our participants’ existing genome-wide data on ~300,000 SNPs measured using the Illumina HumanCytoSNP-12 v2.1 array has been described previously [15,16].

### Arsenic measurements

As previously described [45], arsenic species in HEALS urine samples were separated using high-performance liquid chromatography (HPLC) and detected using inductively coupled plasma-mass spectrometry (ICP-MS) with dynamic reaction cell (DRC). Percentages of iAs, MMA and DMA among all arsenic species were calculated after subtracting arsenobetaine and arsenocholine (i.e., nontoxic organic arsenic from dietary sources) from total arsenic. All data on arsenic species in blood were generated using ICP-MS-DRC coupled to HPLC, as described previously for NIAT and FOX [23,57] (the FACT data is not yet published). Blood samples were processed in the same way for each of these studies, and this processing has been described previously in detail [23] and follows the method of Csanaky and Gregus [58]. For quality control purposes, samples with known concentrations of arsenic species were regularly analyzed. Two samples were run at the beginning of every working day and throughout the day, after every 10 samples, as previously described [23]. The limit of detection for each metabolite of interest was 0.2 μg/L. We have previously reported intra-assay CVs for this assay (from FOX) for As^III^, As^V^, MMA, and DMA (0.9%, 11.5%, 3.6%, and 2.6%, respectively) as well as inter-assay CVs (3.7%, 23.2%, 2.9%, and 3.5%, respectively) [57]. Arsenic exposure in HEALS was assessed by measuring total arsenic concentration in individuals’ urine and their primary drinking well at baseline (2000–2002) [55].

### Statistical methods

We conducted exome-wide association analyses for each of the three arsenic species measured in urine (iAs%, MMA%, and DMA%) restricting to 1,660 HEALS participants with available data on arsenic species in urine. We conducted exome-wide association analyses of arsenical skin lesion status (the most common sign of arsenic toxicity) using data on 2,401 cases and 2,472 lesion-free controls (from both HEALS and BEST). All participants included in these analyses have existing genome-wide data on ~300,000 SNPs based on the Illumina HumanCytoSNP-12 v2.1 array, as described previously [15,16]. For association analysis, we used GEMMA (Genome-wide Efficient Mixed Model Association) [59] to account for cryptic relatedness, as many of our participants have a relative in the study. For the random effects model implemented in GEMMA, we used a kinship matrix based on ~260,000 genome-wide SNPs, as described previously [15]. We also used GEMMA for case/control association testing; we approximated odds ratios (ORs) by first dividing the beta coefficient by [*x*(1 –*x*)], where *x* is the proportion of cases in our sample, in order to estimate the beta from a logistic model. This quantity was exponentiated to obtain an OR.

Multiplicative interaction was tested by including an interaction between arsenic exposure tertiles (coded 0, 1, 2) and rs61735836 (coded 0, 1, or 2 minor alleles) in a logistic regression. Using the results from this logistic regression, additive interaction was estimated as the relative excess risk for interaction (RERI) using the delta method for confidence interval estimation [60,61]. SNP-SNP interaction was tested by including an interaction between two SNPs, coded as minor allele counts, in linear or logistic regression models. In order to analyze the effect of SNPs on arsenic species in blood, including measures taken at multiple time points for the same individuals, we used a mixed-effects model with a random intercept for each individual to account for the fact that 109 individuals appear twice in the dataset (having both baseline and follow-up/post-intervention measurements). Mendelian randomization analyses based on summary statistics were conducted using the inverse-variance weighted meta-analysis method as implemented in the MendelianRandomization R package [44], in addition to a maximum likelihood method, the median methods, and Egger regression [44]. Allele frequencies and linkage disequilibrium (LD) patterns were examined using LDlink [41] and the Geography of Genetic Variants browser [21].

## Supporting information

S1 Fig Cluster plot for rs61735836 (exm1580829). Only two samples did not tightly cluster with one of the three genotype groups. These two were treated as missing.
(GIF)

S2 Fig Exome-wide study of associations between non-synonymous SNPs and arsenic-induced skin lesion status. GWA analyses were conducted using data on 2,401 skin lesion cases and 2,472 lesion-free controls (from both HEALS and BEST) using GEMMA (Genome-wide Efficient Mixed Model Association) to account for cryptic relatedness. Regressions are adjusted for age, sex, and study.
(TIF)

S3 Fig Global allele frequencies for rs61735836. The A/T allele is shown in blue, and the G/C allele is shown in gold. Allele frequency data is from the 1000 Genomes project and the Human Genome Diversity project. Figure generated using the Geography of Genetic Variants (GGV) browser: https://popgen.uchicago.edu/ggv/.
(PDF)

S4 Fig Linkage disequilibrium (LD) values (r^2^) between rs61735836 and surrounding variants in 1KG populations. The blue dot represent the LD between rs61735836 and itself (i.e., r^2^ = 1). BEB, Bengali from Bangladesh; SAS, South Asian super-population; AFR, African super-population; AMR, American super-population; EAS, East Asian super-population. Figures generated using LDlink (https://analysistools.nci.nih.gov/LDlink/)
(PDF)

S5 Fig Expression of FTCD in human tissues from the GTEx (Genotype-Tissue Expression) Project. Top: Expression shown in TPM (transcripts per kilobase million). Bottom: Expression shown as log_10_(TPM).
(PDF)

S6 Fig *FTCD* missense variant rs61735836 creates the first 5´-proximal canonical Kozak sequence ([A/G]xxAUGG) in the *FTCD* gene. Of the three start codons that are 5´ to rs61735836, none are canonical Kozak sequences.
(PDF)

S7 Fig ENCODE annotations indicative of regulatory elements in the *FTCD* region. Exon 3 of *FTCD* (containing rs61735836) resides in a “weak promoter” (based on HepG2 chromatin state segmentation HMM) as lies down stream of various putative transcript factor binding sides, DNaseI hypersensitivity sites, and histone marks indicative of regulatory elements (based on various ENCODE cell lines).
(PDF)

S8 Fig Exon expression for FTCD. Inferred isoforms and TPM (transcripts per kilobase million) based on GTEx Analysis Release v7.
(TIF)

S9 Fig Histograms of urinary FIGLU (creatinine-adjusted) for each of the three genotype categories for s61735836. FIGLU was measured for 15, 14, and 15 HEALS participants within each genotype category.
(TIF)

S10 Fig Mendelian randomization analyses support a causal effect of arsenic metabolism efficiency (as measured by DMA%, MMA%, or iAs%) on arsenic-induced skin lesions. All methods reported in tables (left) are implemented in the MendelianRandomization R package. Scatterplots (right) show horizontal and vertical error bars corresponding to the 95% CI for the beta coefficient for each SNP’s association with arsenic species percentage in urine (DMA%, MMA%, or iAs%) and skin lesion risk, respectively. The slope of the diagonal line is the inverse-variance-weighted (IVW) estimate of the causal effect (i.e., the ln(OR).
(PDF)

S1 Table Associations between the minor allele of *FTCD* SNP rs61735836 (A) and arsenic metabolism phenotypes (n = 1,660). (PDF)

S2 Table Associations between the minor allele of *FTCD* SNP rs61735836 (A) and arsenic metabolism phenotypes, stratified by sex and median age (n = 1,660). (PDF)

S3 Table SNP-SNP interactions for three arsenic metabolism-related SNPs in relation to urinary DMA% and skin lesion status. (PDF)

S4 Table Associations between the minor alleles at FTCD and AS3MT SNPs with arsenic species percentages measured in blood at two time points (n = 155). (PDF)

S1 File Summary statistics for an exome-wide association study of urinary DMA%. (TXT)

S2 File Summary statistics for an exome-wide association study of urinary MMA%. (TXT)

S3 File Summary statistics for an exome-wide association study of urinary iAs%. (TXT)

S4 File Summary statistics for an exome-wide association study of arsenic-induced skin lesion status. (TXT)
