# Correction: A missense variant in *FTCD* is associated with arsenic metabolism and toxicity phenotypes in Bangladesh

**DOI:** 10.1371/journal.pgen.1008172

**Published:** 2019-05-20

**Authors:** Brandon L. Pierce, Lin Tong, Samantha Dean, Maria Argos, Farzana Jasmine, Muhammad Rakibuz-Zaman, Golam Sarwar, Md. Tariqul Islam, Hasan Shahriar, Tariqul Islam, Mahfuzar Rahman, Md. Yunus, Vincent J. Lynch, Devin Oglesbee, Joseph H. Graziano, Muhammad G. Kibriya, Mary V. Gamble, Habibul Ahsan

There is an error in the third sentence of the second paragraph within the Results/Discussion section. The genotypes for s61735836 should be listed as ‘AA’ and ‘AG’, rather than ‘AA’ and ‘AC’. The corrected version of this sentence is: “Individuals carrying two minor alleles (AA) as compared to one (AG) appear to have even lower DMA%, suggesting a potential additive effect of the A allele; however, our sample size of minor allele homozygotes was small (n = 12), limiting our ability to examine differences between these two groups (S1 Table).”

Additionally, S1 Table also contains the incorrectly labelled genotypes ‘AC’ (which should be ‘AG’) and ‘CC’ (which should be ‘GG’). A corrected version is provided below.

In S4 Table, the minor allele is referred to as ‘T’, which is inconsistent with how the authors refer to this allele in the remainder of the article (i.e., it is the allele on the opposite strand). The minor allele should be ‘A’. A corrected version is provided below.

## Supporting information

S1 Table Associations between the minor allele of *FTCD* SNP rs61735836 (A) and arsenic metabolism phenotypes (n = 1,660). (DOCX)

S4 Table Associations between the minor alleles at FTCD and AS3MT SNPs with arsenic species percentages measured in blood at two time points (n = 155). (DOCX)
