# Supplementary figures and images for: A missense variant in FTCD is associated with arsenic metabolism and toxicity phenotypes in Bangladesh
Source: PLoS Genet. 2019 Mar 20;15(3):e1007984. doi: 10.1371/journal.pgen.1007984 (PMC6443193; doi:10.1371/journal.pgen.1007984)

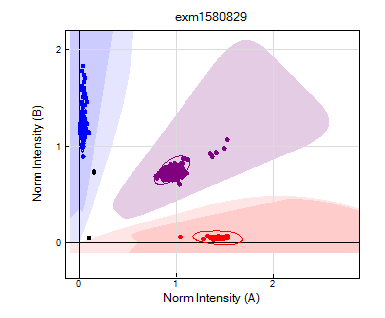

Supplement: S1 Fig — Only two samples did not tightly cluster with one of the three genotype groups. These two were treated as missing. (GIF) [file pgen.1007984.s001.gif]

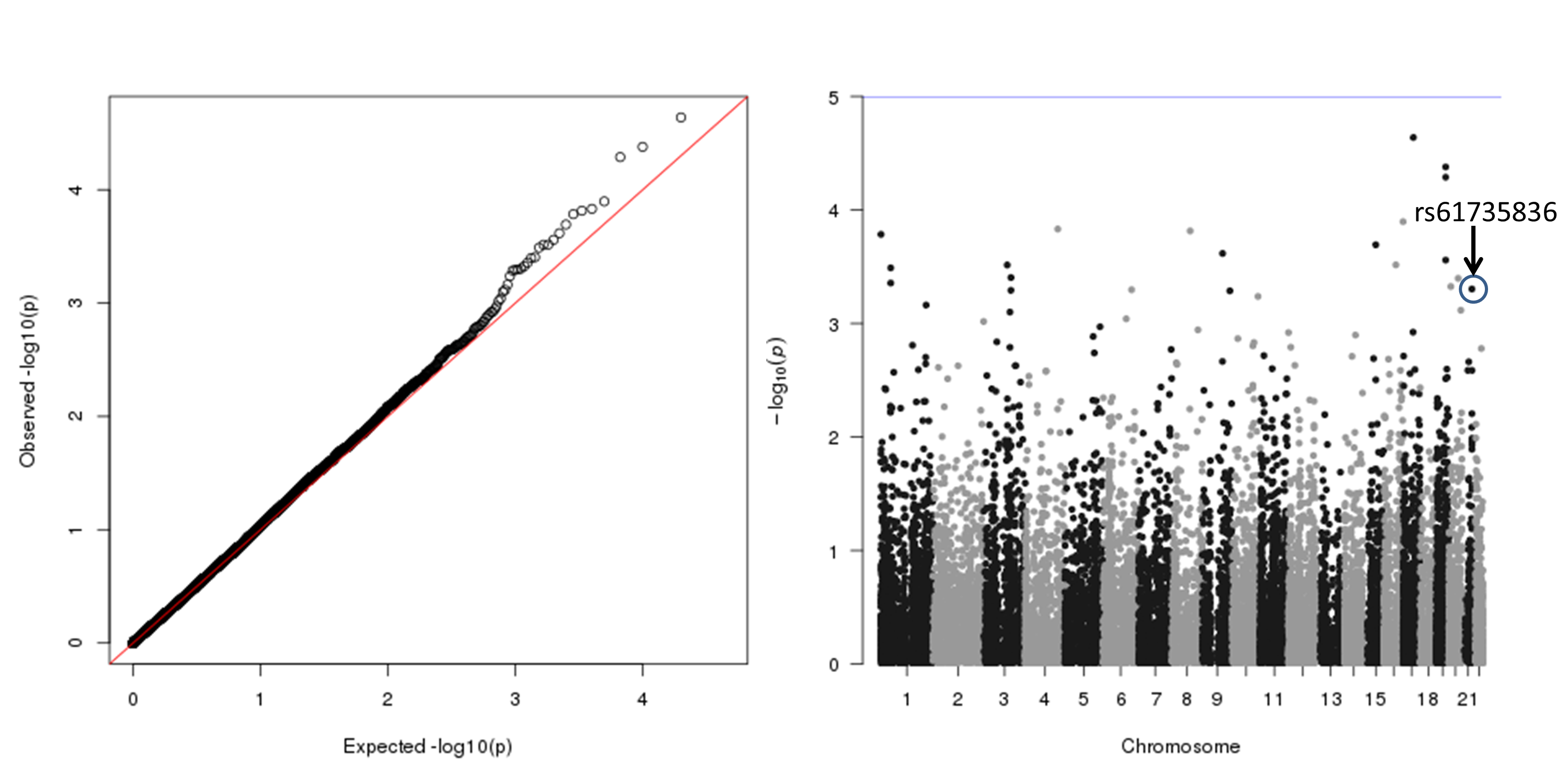

Supplement: S2 Fig — GWA analyses were conducted using data on 2,401 skin lesion cases and 2,472 lesion-free controls (from both HEALS and BEST) using GEMMA (Genome-wide Efficient Mixed Model Association) to account for cryptic relatedness. Regressions are adjusted for age, sex, and study. (TIF) [file pgen.1007984.s002.tif]

ENSG00000160282.9 Gene Expression

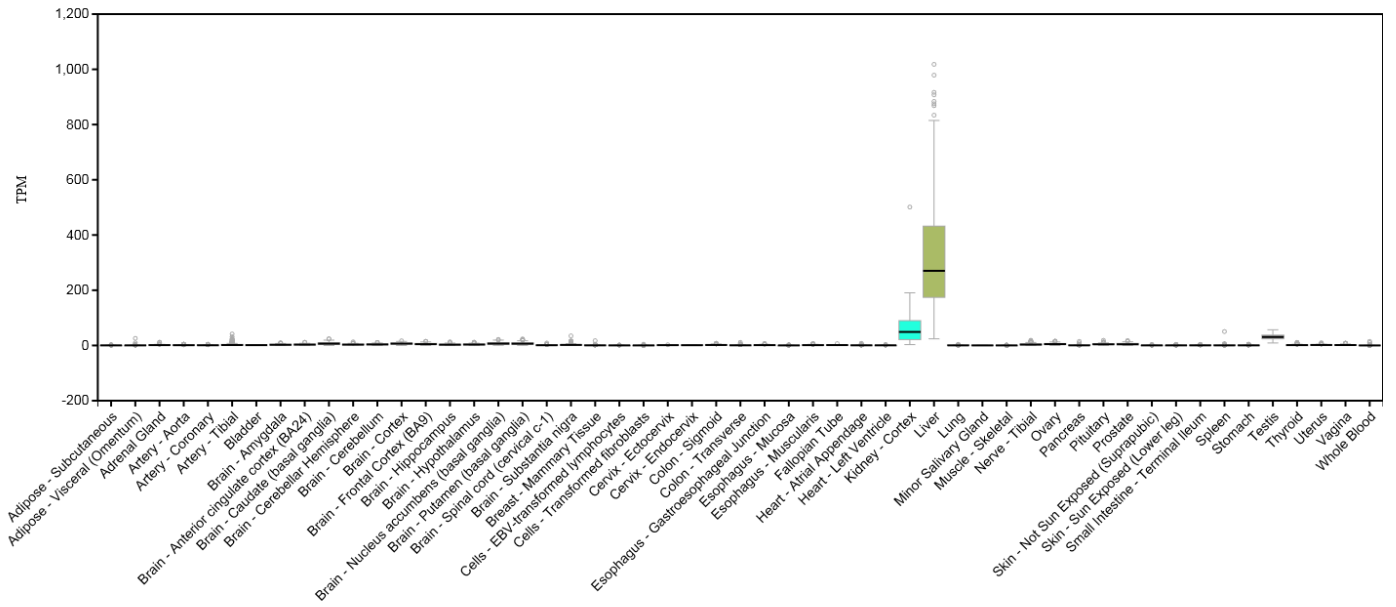

ENSG00000160282.9 Gene Expression

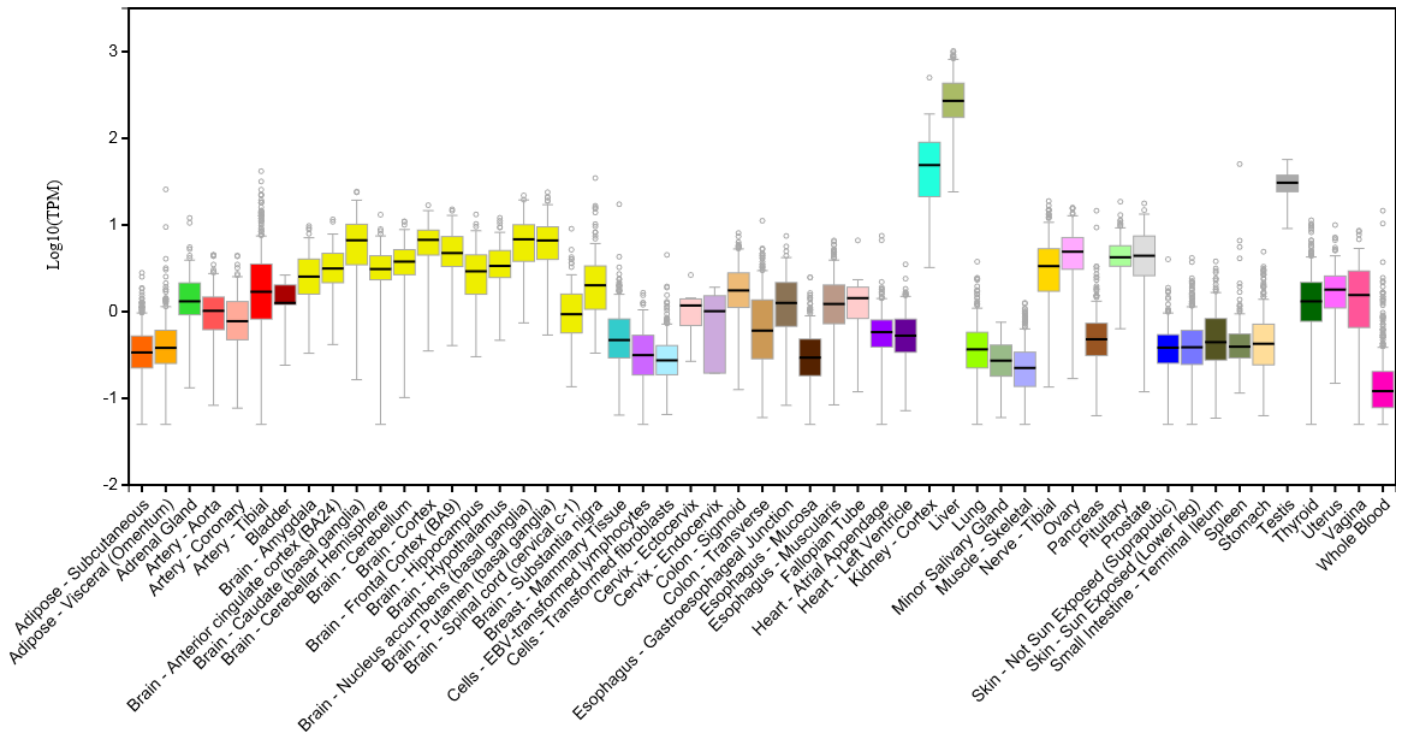

Supplement: S5 Fig — Top: Expression shown in TPM (transcripts per kilobase million). Bottom: Expression shown as log10(TPM). (PDF) [file pgen.1007984.s005.pdf]

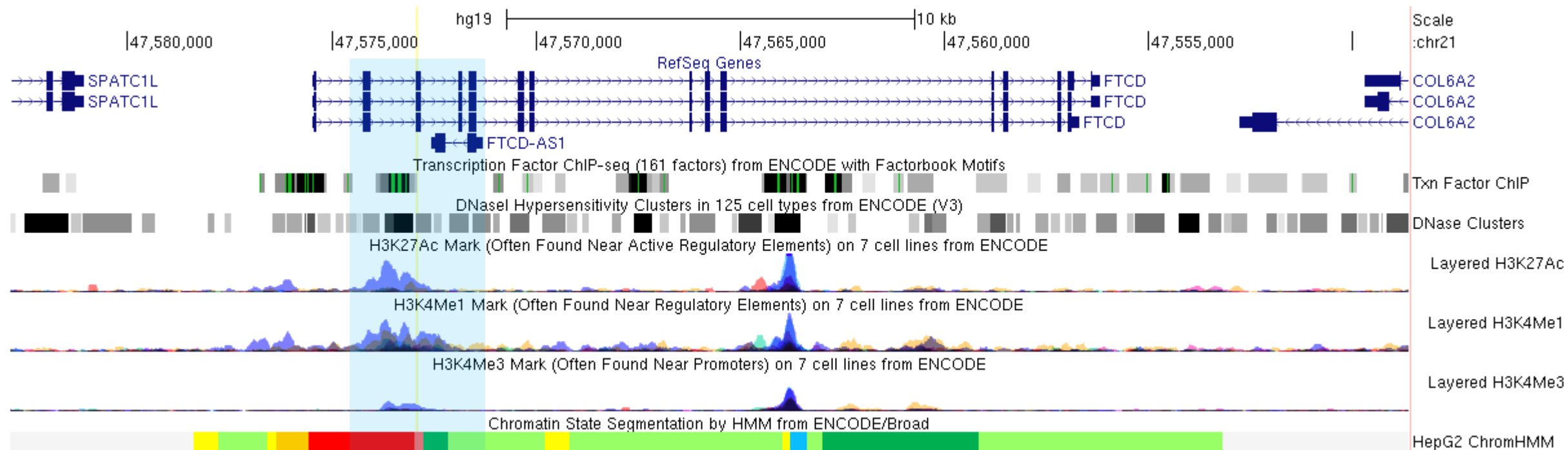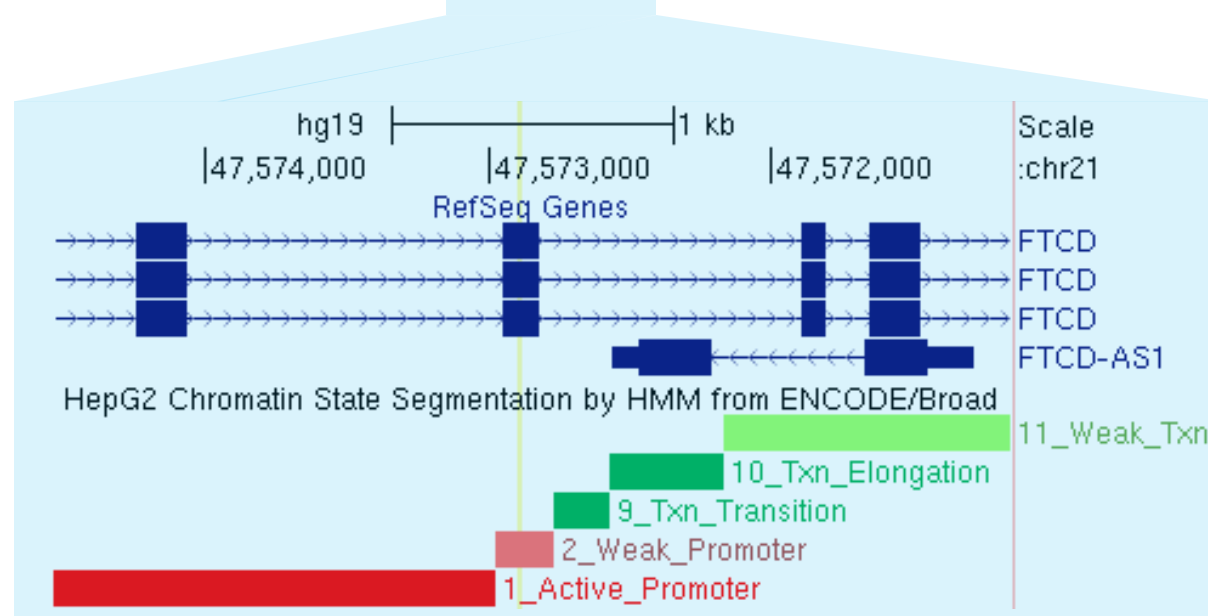

Supplement: S7 Fig — Exon 3 of FTCD (containing rs61735836) resides in a “weak promoter” (based on HepG2 chromatin state segmentation HMM) as lies down stream of various putative transcript factor binding sides, DNaseI hypersensitivity sites, and histone marks indicative of regulatory elements (based on various ENCODE cell lines). (PDF) [file pgen.1007984.s007.pdf]

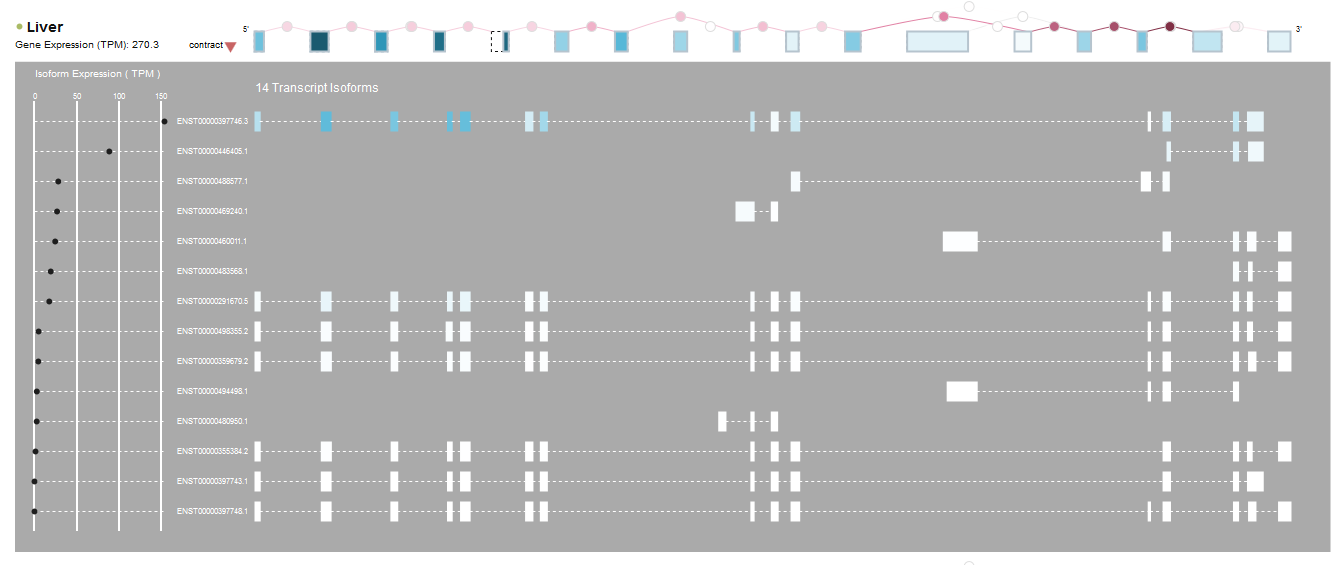

Supplement: S8 Fig — Inferred isoforms and TPM (transcripts per kilobase million) based on GTEx Analysis Release v7. (TIF) [file pgen.1007984.s008.tif]

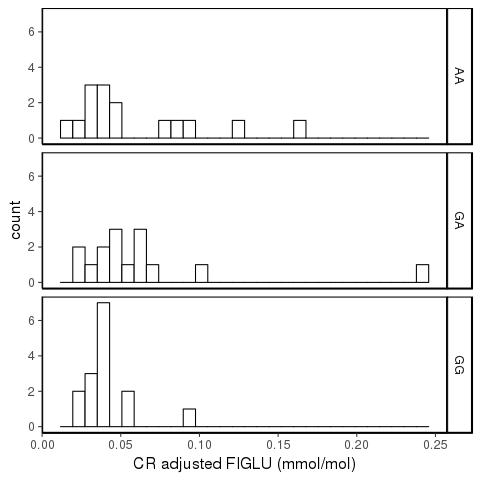

Supplement: S9 Fig — FIGLU was measured for 15, 14, and 15 HEALS participants within each genotype category. (TIF) [file pgen.1007984.s009.tif]
