# Supplementary material for: A missense variant in FTCD is associated with arsenic metabolism and toxicity phenotypes in Bangladesh
Source: PLoS Genet. 2019 Mar 20;15(3):e1007984. doi: 10.1371/journal.pgen.1007984 (PMC6443193; doi:10.1371/journal.pgen.1007984)

chr21:47572887 T/C

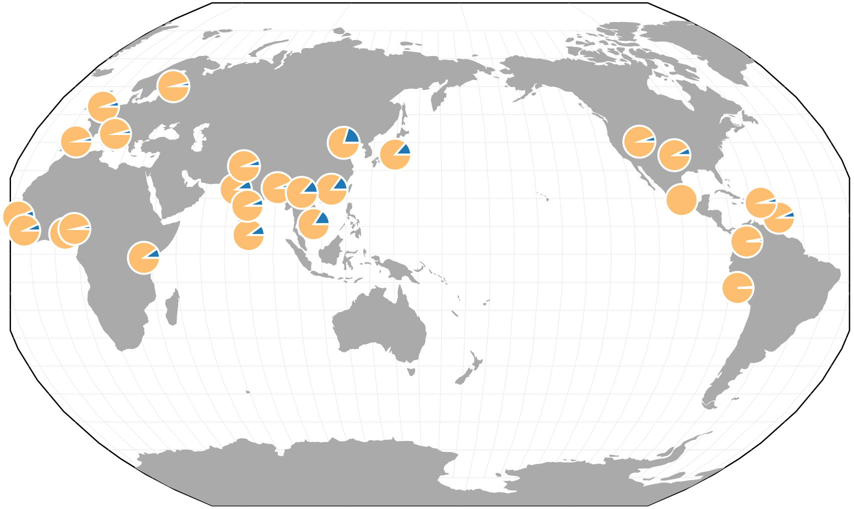

Frequency Scale = Proportion out of 1
The pie below represents a minor allele frequency of 0.25

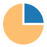

Sample sizes below 30 become increasingly transparent to represent uncertain frequencies, i.e.

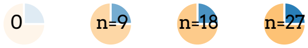

Supplement: S3 Fig — The A/T allele is shown in blue, and the G/C allele is shown in gold. Allele frequency data is from the 1000 Genomes project and the Human Genome Diversity project. Figure generated using the Geography of Genetic Variants (GGV) browser: https://popgen.uchicago.edu/ggv/. (PDF) [file pgen.1007984.s003.pdf]
