# Supplementary material for: A missense variant in FTCD is associated with arsenic metabolism and toxicity phenotypes in Bangladesh
Source: PLoS Genet. 2019 Mar 20;15(3):e1007984. doi: 10.1371/journal.pgen.1007984 (PMC6443193; doi:10.1371/journal.pgen.1007984)
Supplement: S4 Fig — The blue dot represent the LD between rs61735836 and itself (i.e., r2 = 1). BEB, Bengali from Bangladesh; SAS, South Asian super-population; AFR, African super-population; AMR, American super-population; EAS, East Asian super-population. Figures generated using LDlink (https://analysistools.nci.nih.gov/LDlink/) (PDF) [file pgen.1007984.s004.pdf]

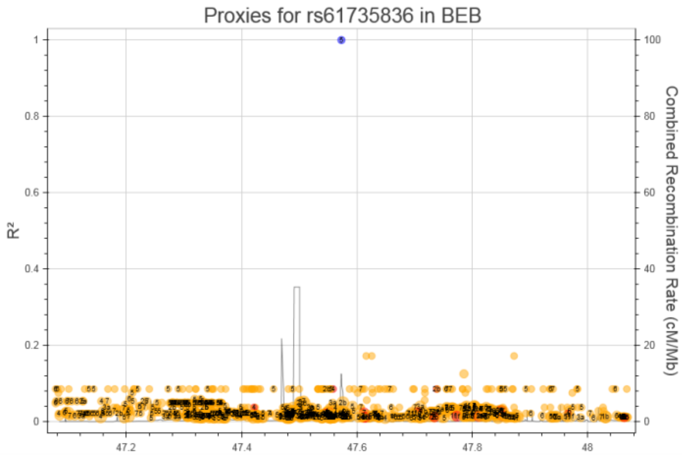
Proxies for rs61735836 in BEB
R²
Combined Recombination Rate (cM/Mb)

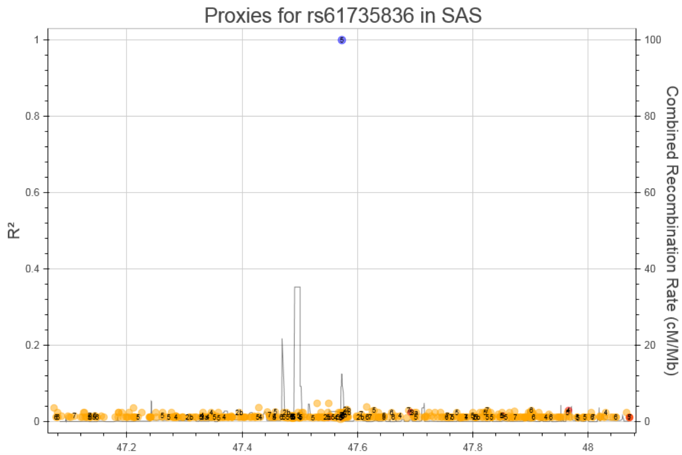
Proxies for rs61735836 in SAS
R²
Combined Recombination Rate (cM/Mb)

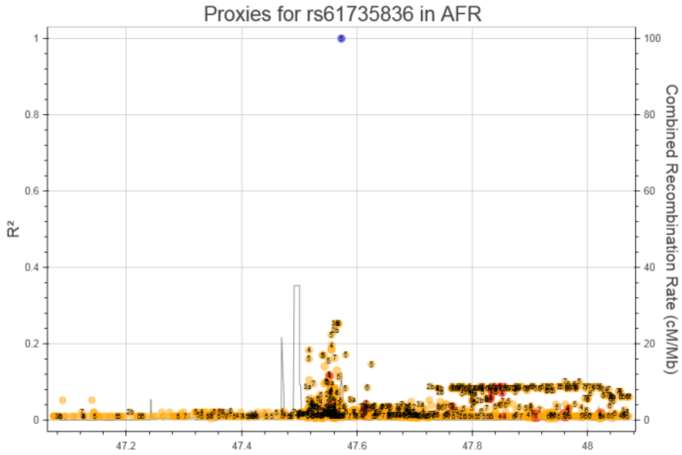
Proxies for rs61735836 in AFR
R²
Combined Recombination Rate (cM/Mb)

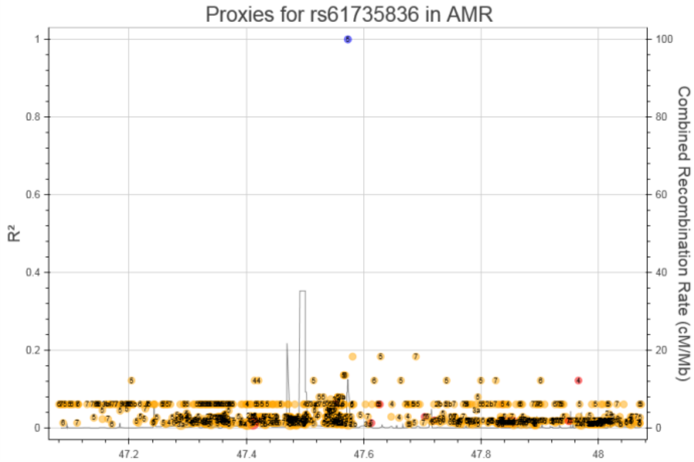
Proxies for rs61735836 in AMR
R²
Combined Recombination Rate (cM/Mb)

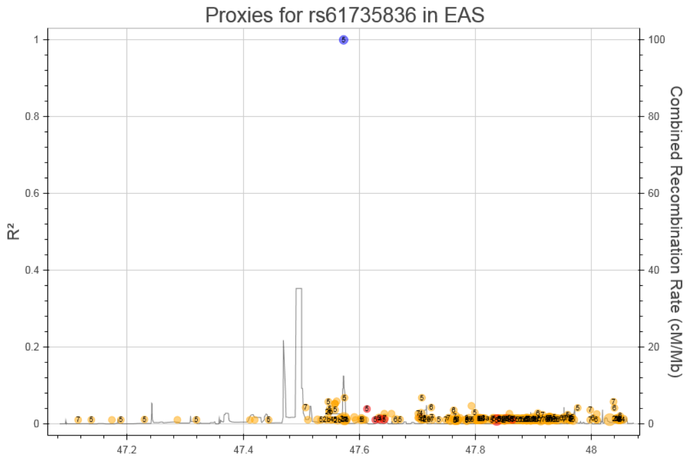
Proxies for rs61735836 in EAS
R²
Combined Recombination Rate (cM/Mb)

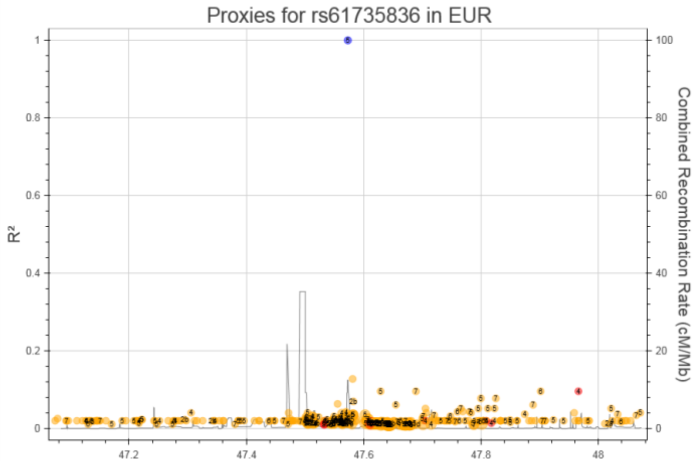
Proxies for rs61735836 in EUR
R²
Combined Recombination Rate (cM/Mb)
