# Supplementary material for: A missense variant in FTCD is associated with arsenic metabolism and toxicity phenotypes in Bangladesh
Source: PLoS Genet. 2019 Mar 20;15(3):e1007984. doi: 10.1371/journal.pgen.1007984 (PMC6443193; doi:10.1371/journal.pgen.1007984)

**The first AUG** is the canonical start codon, but this sequence does not have a G following the start codon (GCCAUGU).

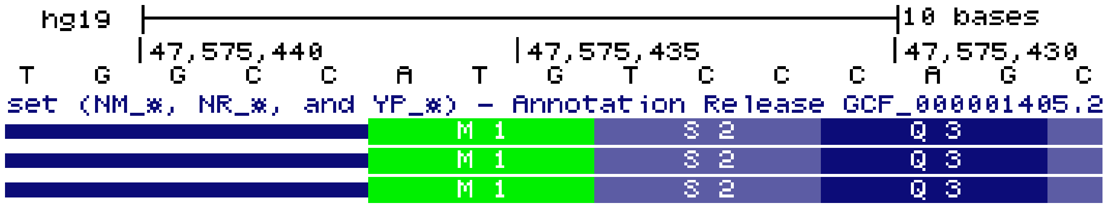


**The second AUG** is in exon 2 which also does not have a G following the start codon (GACAUGA).

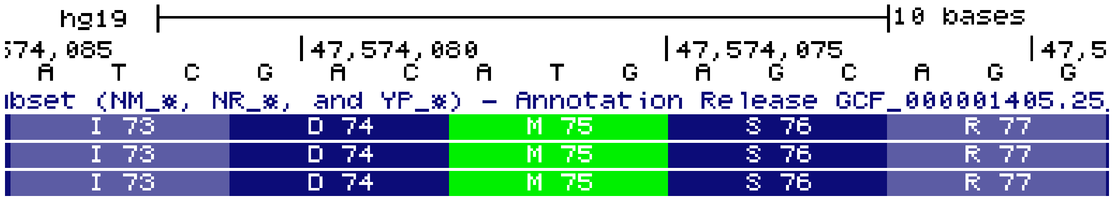


**The third AUG** is in exon 3, which does not have an A or a G three bp upstream of the start codon (CGCAUGG).

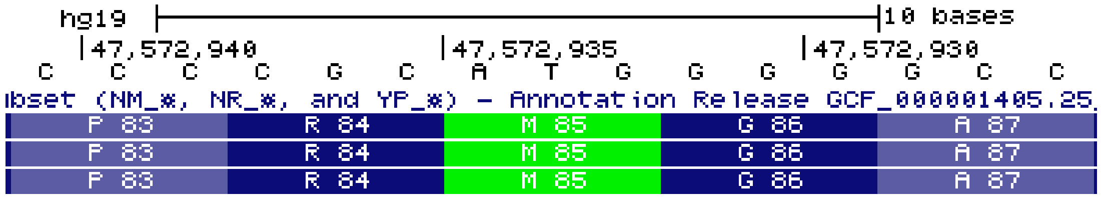

Supplement: S6 Fig — Of the three start codons that are 5´ to rs61735836, none are canonical Kozak sequences. (PDF) [file pgen.1007984.s006.pdf]
