# Supplementary material for: A missense variant in FTCD is associated with arsenic metabolism and toxicity phenotypes in Bangladesh
Source: PLoS Genet. 2019 Mar 20;15(3):e1007984. doi: 10.1371/journal.pgen.1007984 (PMC6443193; doi:10.1371/journal.pgen.1007984)

Mendelian randomization results for DMA%

Method	Estimate	SE	P-value
Max likelihood	-0.013	0.003	4.5E-7
Simple median	-0.011	0.003	7.1E-04
Weighted median	-0.011	0.003	5.9E-05
IVW	-0.013	0.002	6.7E-08
MR-Egger	-0.011	0.008	0.170
MR-Egger (intercept)	-0.006	0.029	0.832

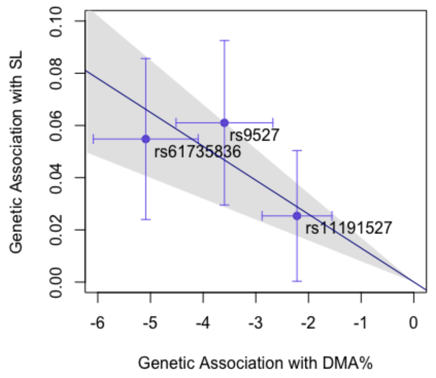

Mendelian randomization results for MMA%

Method	Estimate	SE	P-value
Max likelihood	0.026	0.005	6.8E-7
Simple median	0.026	0.007	1.8E-04
Weighted median	0.025	0.006	1.4E-05
IVW	0.026	0.005	4.6E-08
MR-Egger	0.023	0.013	0.076
MR-Egger (intercept)	0.005	0.023	0.838

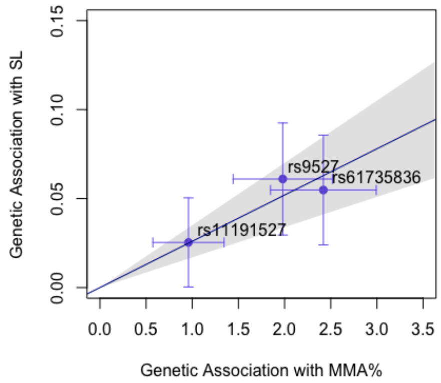

Mendelian randomization results for iAs%

Method	Estimate	SE	P-value
Max likelihood	0.024	0.005	2.9E-6
Simple median	0.020	0.007	0.006
Weighted median	0.020	0.006	6.6E-04
IVW	0.024	0.005	1.2E-07
MR-Egger	0.017	0.021	0.414
MR-Egger (intercept)	0.014	0.038	0.716

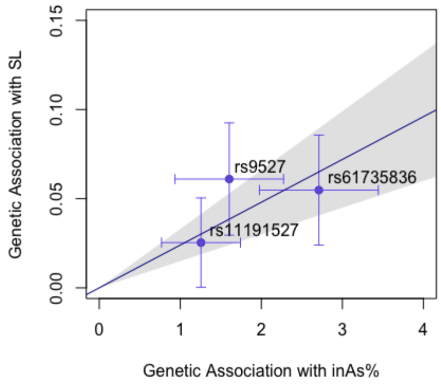

Supplement: S10 Fig — All methods reported in tables (left) are implemented in the MendelianRandomization R package. Scatterplots (right) show horizontal and vertical error bars corresponding to the 95% CI for the beta coefficient for each SNP’s association with arsenic species percentage in urine (DMA%, MMA%, or iAs%) and skin lesion risk, respectively. The slope of the diagonal line is the inverse-variance-weighted (IVW) estimate of the causal effect (i.e., the ln(OR). (PDF) [file pgen.1007984.s010.pdf]
