# Supplementary material for: A missense variant in FTCD is associated with arsenic metabolism and toxicity phenotypes in Bangladesh
Source: PLoS Genet. 2019 Mar 20;15(3):e1007984. doi: 10.1371/journal.pgen.1007984 (PMC6443193; doi:10.1371/journal.pgen.1007984)
Supplement: S1 Table — (PDF) [file pgen.1007984.s011.pdf]

S1 Table. Associations[a] between the minor allele of *FTCD* SNP rs61735836 (A) and arsenic metabolism phenotypes (n=1,660)

	Per-allele association			AC vs. CC			AA vs. CC		
	**Beta**	**SE**	**P-value**	**Beta**	**SE**	**P-value**	**Beta**	**SE**	**P-value**
**Arsenic metabolites**									
DMA%	-5.09	0.51	5.76E-23	-4.86	0.57	1.29E-17	-6.05	1.15	1.52E-07
MMA%	2.42	0.29	2.16E-16	2.28	0.32	2.22E-12	3.03	0.65	3.71E-06
iAs%	2.71	0.37	7.56E-13	2.63	0.42	2.46E-10	3.04	0.85	3.26E-04
ln(total Arsenic)[b]	0.07	0.07	3.19E-01	0.08	0.08	0.29	0.02	0.15	0.89
ln(DMA)[b,c]	-0.08	0.007	3.14E-24	-0.06	0.01	1.19E-12	-0.08	0.02	7.33E-05
ln(MMA)[b,c]	0.18	0.02	2.21E-14	0.17	0.03	1.60E-11	0.20	0.05	1.92E-04
ln(iAs)[b,c]	0.19	0.02	7.30E-14	0.19	0.03	7.16E-12	0.18	0.06	1.81E-03
**Latent Phenotypes (PCA)**[d]									
PC1	0.91	0.09	1.16E-23	0.88	0.10	5.26E-19	1.08	0.20	6.56E-08
PC2	0.06	0.06	3.21E-01	0.03	0.06	0.59	0.11	0.12	0.39
**Methylation indices**[e]									
PMI	-0.03	0.04	4.92E-01	-0.04	0.05	0.42	-0.01	0.10	0.90
SMI	-1.51	0.22	3.88E-12	-1.55	0.24	1.21E-10	-1.42	0.50	4.72E-03

[a] associations estimated using a linear mixed model adjusting for age, sex, and relatedness in GEMMA
[b] phenotypes are natural log-transformed to reduce skewness
[c] phenotypes are raw metabolite concentrations, not percentages of total arsenic. Regressions are adjusted for total arsenic.
[d] phenotypes are latent variables obtained from principle components analysis (PCA) of all three metabolite percentages.
[e] PMI (primary methylation index) = MMA/iAs; SMI (secondary methylation index) = DMA/MMA
[f] number of participants with rs61735836 genotypes of CC, AC, AA were 1417, 231 and 12 respectively.
