# Supplementary material for: A missense variant in FTCD is associated with arsenic metabolism and toxicity phenotypes in Bangladesh
Source: PLoS Genet. 2019 Mar 20;15(3):e1007984. doi: 10.1371/journal.pgen.1007984 (PMC6443193; doi:10.1371/journal.pgen.1007984)
Supplement: S2 Table — (PDF) [file pgen.1007984.s012.pdf]

S2 Table. Associations[a] between the minor allele of *FTCD* SNP rs61735836 (A) and arsenic metabolism phenotypes, stratified by sex and median age (n=1,660)

	**Male**			**Female**			**≤37 years of age**			**>37 years of age**		
	**Beta**	**SE**	**P-value**	**Beta**	**SE**	**P-value**	**Beta**	**SE**	**P-value**	**Beta**	**SE**	**P-value**
**Arsenic species**												
DMA%	-5.31	0.7	2.39E-14	-4.94	0.77	1.08E-10	-4.82	0.77	3.09E-10	-5.65	0.78	4.65E-13
MMA%	2.58	0.43	1.91E-09	2.23	0.41	6.15E-08	1.97	0.44	7.63E-06	3.01	0.48	2.94E-10
iAs%	2.75	0.48	8.11E-09	2.78	0.57	1.06E-06	2.85	0.56	2.85E-07	2.63	0.53	6.94E-07
ln(total Arsenic)[b]	-0.07	0.1	4.55E-01	0.21	0.1	3.01E-02	0.21	0.1	3.30E-02	-0.04	0.1	6.73E-01
**Latent Phenotypes (PCA)** [c]												
PC1	0.94	0.12	2.26E-14	0.89	0.13	2.57E-11	0.85	0.14	3.08E-10	1.01	0.14	2.23E-13
PC2	0.07	0.08	3.26E-01	0.02	0.08	7.86E-01	-0.03	0.08	6.85E-01	0.15	0.08	8.25E-02
**Methylation indices** [d]												
PMI	-0.02	0.04	6.65E-01	-0.01	0.04	7.94E-01	-0.05	0.04	1.95E-01	0.03	0.04	5.58E-01
SMI	-1.18	0.2	4.53E-09	-1.69	0.32	1.28E-07	-1.35	0.31	1.59E-05	-1.61	0.28	5.70E-09

[a] associations estimated using a linear mixed model adjusting for age, sex, and relatedness in GEMMA
[b] natural log-transformed to reduce skewness
[c] phenotypes are latent variables obtained from principle components analysis (PCA) of all three metabolite percentages.
[d] PMI (primary methylation index) = MMA/iAs; SMI (secondary methylation index) = DMA/MMA
