# Supplementary material for: A missense variant in FTCD is associated with arsenic metabolism and toxicity phenotypes in Bangladesh
Source: PLoS Genet. 2019 Mar 20;15(3):e1007984. doi: 10.1371/journal.pgen.1007984 (PMC6443193; doi:10.1371/journal.pgen.1007984)
Supplement: S3 Table — (PDF) [file pgen.1007984.s013.pdf]

S3 Table. SNP-SNP interactions between FTCD SNP rs61735836 and two AS3MT SNPs (rs9537 and rs11191527) in relation to urinary DMA% and skin lesion status

Models	Outcome	Parameter	Estimate/OR	SE	p-value
Model 1	DMA%	rs9527	-3.57	0.56	2.36E-10
	DMA%	rs61735836	-5.35	0.54	2.79E-22
	DMA%	rs9527*rs61735836	1.34	1.28	0.30
Model 2	DMA%	rs11191527	1.94	0.40	1.78E-06
	DMA%	rs61735836	-5.30	0.59	8.52E-12
	DMA%	rs1119152*rs61735836	0.55	0.96	0.85
Model 3	Skin Lesion	rs9527	1.54	0.09	1.10E-06
	Skin Lesion	rs61735836	1.49	0.09	1.04E-05
	Skin Lesion	rs9527*rs61735836	0.79	0.20	0.25
Model 4	Skin Lesion	rs11191527	0.87	0.07	0.05
	Skin Lesion	rs61735836	1.41	0.10	3.82E-04
	Skin Lesion	rs1119152*rs61735836	1.05	0.16	0.76

n=1,660 for Model 1-2; n=4,873 for Model 4-6 (2,401 skin lesion cases, 2,472 controls). Regression models were adjusted for age, sex, genotyping batch and the AS3MT SNP not included in the interaction term (rs9537 or rs11191527). SNPs were coded as minor allele counts.
