# Supplementary material for: A missense variant in FTCD is associated with arsenic metabolism and toxicity phenotypes in Bangladesh
Source: PLoS Genet. 2019 Mar 20;15(3):e1007984. doi: 10.1371/journal.pgen.1007984 (PMC6443193; doi:10.1371/journal.pgen.1007984)
Supplement: S4 Table — (PDF) [file pgen.1007984.s014.pdf]

S4 Table. Associations[a] between the minor alleles[b] at FTCD and AS3MT SNPs with arsenic species percentages measured in blood at two time points (n=155)

Outcome	rs61735836 (FTCD)			rs9527 (AS3MT)			rs11191527 (AS3MT)		
	Beta	SE	P-value	Beta	SE	P-value	Beta	SE	P-value
DMA% at Week 0	-2.92	1.22	0.02	-1.59	1.22	0.19	1.25	0.87	0.15
DMA% at Week 12	-2.80	1.82	0.13	-1.17	1.79	0.51	1.09	1.26	0.39
DMA% at Weeks 0 and 12	-2.87	1.01	0.005	-1.46	1.00	0.15	1.21	0.71	0.09
InAs% at Week 0	1.90	0.79	0.02	0.03	0.80	0.97	-0.02	0.58	0.98
InAs% at Week 12	-1.03	1.18	0.39	-0.24	1.15	0.83	-0.62	0.81	0.45
InAs% at Weeks 0 and 12	0.96	0.66	0.15	-0.06	0.65	0.93	-0.22	0.47	0.64
MMA% at Week 0	1.02	1.22	0.41	1.56	1.20	0.20	-1.24	0.86	0.15
MMA% at Week 12	3.83	1.35	0.01	1.42	1.36	0.30	-0.48	0.96	0.62
MMA% at Weeks 0 and 12	2.19	0.91	0.02	1.50	0.90	0.10	-0.93	0.64	0.15
Total blood As[c] at Week 0	0.08	0.13	0.57	-0.02	0.13	0.88	-0.12	0.09	0.20
Total blood As[c] at Week 12	0.28	0.14	0.06	0.06	0.14	0.66	-0.08	0.10	0.44
Total blood As[c] at Weeks 0 and 12	0.15	0.10	0.11	0.01	0.10	0.88	-0.10	0.07	0.14

[a] Linear models adjusting for age, sex and genotyping batch were used for analyses of Week 0 and Week 12 data. Linear mixed models adjusting for age, sex and genotyping batch, random effect of week/timepoint were used for combined analysis of Week 0 and Week 12 blood arsenic data. Models for rs11191527 were also adjusted for rs9527, and vice versa.

[b] The minor alleles of the three SNPs were all T. The MAF for rs61735836 is 8%, the MAF for rs9527 is 7% and the MAF for rs11191527 is 15%.

[c] Total blood As is the sum of blood AsIII, AsV, MMA and DMA measurements.
