# Supplementary material for: Correction: A missense variant in FTCD is associated with arsenic metabolism and toxicity phenotypes in Bangladesh
Source: PLoS Genet. 2019 May 20;15(5):e1008172. doi: 10.1371/journal.pgen.1008172 (PMC6527204; doi:10.1371/journal.pgen.1008172)
Supplement: S1 Table — (DOCX) [file pgen.1008172.s001.docx]

**S1 Table. Associations^a^ between the minor allele of *FTCD* SNP rs61735836 (A) and arsenic metabolism phenotypes (n=1,660)**

|  | **Per-allele association** | | | **AG vs. GG** | | | **AA vs. GG** | | |
| --- | --- | --- | --- | --- | --- | --- | --- | --- | --- |
|  | **Beta** | **SE** | **P-value** | **Beta** | **SE** | **P-value** | **Beta** | **SE** | **P-value** |
| **Arsenic metabolites** |  |  |  |  |  |  |  |  |  |
| DMA% | -5.09 | 0.51 | 5.76E-23 | -4.86 | 0.57 | 1.29E-17 | -6.05 | 1.15 | 1.52E-07 |
| MMA% | 2.42 | 0.29 | 2.16E-16 | 2.28 | 0.32 | 2.22E-12 | 3.03 | 0.65 | 3.71E-06 |
| iAs% | 2.71 | 0.37 | 7.56E-13 | 2.63 | 0.42 | 2.46E-10 | 3.04 | 0.85 | 3.26E-04 |
| ln(total Arsenic)^b^ | 0.07 | 0.07 | 3.19E-01 | 0.08 | 0.08 | 0.29 | 0.02 | 0.15 | 0.89 |
| ln(DMA)^b,c^ | -0.08 | 0.007 | 3.14E-24 | -0.06 | 0.01 | 1.19E-12 | -0.08 | 0.02 | 7.33E-05 |
| ln(MMA) ^b,c^ | 0.18 | 0.02 | 2.21E-14 | 0.17 | 0.03 | 1.60E-11 | 0.20 | 0.05 | 1.92E-04 |
| ln(iAs) ^b,c^ | 0.19 | 0.02 | 7.30E-14 | 0.19 | 0.03 | 7.16E-12 | 0.18 | 0.06 | 1.81E-03 |
| **Latent Phenotypes (PCA)** ^d^ |  |  |  |  |  |  |  |  |  |
| PC1 | 0.91 | 0.09 | 1.16E-23 | 0.88 | 0.10 | 5.26E-19 | 1.08 | 0.20 | 6.56E-08 |
| PC2 | 0.06 | 0.06 | 3.21E-01 | 0.03 | 0.06 | 0.59 | 0.11 | 0.12 | 0.39 |
| **Methylation indices** ^e^ |  |  |  |  |  |  |  |  |  |
| PMI | -0.03 | 0.04 | 4.92E-01 | -0.04 | 0.05 | 0.42 | -0.01 | 0.10 | 0.90 |
| SMI | -1.51 | 0.22 | 3.88E-12 | -1.55 | 0.24 | 1.21E-10 | -1.42 | 0.50 | 4.72E-03 |

^a^ associations estimated using a linear mixed model adjusting for age, sex, and relatedness in GEMMA

^b^ phenotypes are natural log-transformed to reduce skewness

^c^  phenotypes are raw metabolite concentrations, not percentages of total arsenic. Regressions are adjusted for total arsenic.

^d^ phenotypes are latent variables obtained from principle components analysis (PCA) of all three metabolite percentages.

^e^ PMI (primary methylation index) = MMA/iAs; SMI (secondary methylation index) = DMA/MMA

^f^ number of participants with rs61735836 genotypes of GG, AG, AA were 1417, 231 and 12 respectivel
