# Supplementary material for: Correction: A missense variant in FTCD is associated with arsenic metabolism and toxicity phenotypes in Bangladesh
Source: PLoS Genet. 2019 May 20;15(5):e1008172. doi: 10.1371/journal.pgen.1008172 (PMC6527204; doi:10.1371/journal.pgen.1008172)
Supplement: S4 Table — (DOCX) [file pgen.1008172.s002.docx]

**S4 Table. Associations^a^ between the minor alleles^b^ at FTCD and AS3MT SNPs with arsenic species percentages measured in blood at two time points (n=155)**

| **Outcome** | **rs61735836 (FTCD)** | | | **rs9527 (AS3MT)** | | | **rs11191527 (AS3MT)** | | |
| --- | --- | --- | --- | --- | --- | --- | --- | --- | --- |
|  | **Beta** | **SE** | **P-value** | **Beta** | **SE** | **P-value** | **Beta** | **SE** | **P-value** |
| **DMA% at Week 0** | -2.92 | 1.22 | 0.02 | -1.59 | 1.22 | 0.19 | 1.25 | 0.87 | 0.15 |
| **DMA% at Week 12** | -2.80 | 1.82 | 0.13 | -1.17 | 1.79 | 0.51 | 1.09 | 1.26 | 0.39 |
| **DMA% at Weeks 0 and 12** | -2.87 | 1.01 | 0.005 | -1.46 | 1.00 | 0.15 | 1.21 | 0.71 | 0.09 |
|  |  |  |  |  |  |  |  |  |  |
| **InAs% at Week 0** | 1.90 | 0.79 | 0.02 | 0.03 | 0.80 | 0.97 | -0.02 | 0.58 | 0.98 |
| **InAs% at Week 12** | -1.03 | 1.18 | 0.39 | -0.24 | 1.15 | 0.83 | -0.62 | 0.81 | 0.45 |
| **InAs% at Weeks 0 and 12** | 0.96 | 0.66 | 0.15 | -0.06 | 0.65 | 0.93 | -0.22 | 0.47 | 0.64 |
|  |  |  |  |  |  |  |  |  |  |
| **MMA% at Week 0** | 1.02 | 1.22 | 0.41 | 1.56 | 1.20 | 0.20 | -1.24 | 0.86 | 0.15 |
| **MMA% at Week 12** | 3.83 | 1.35 | 0.01 | 1.42 | 1.36 | 0.30 | -0.48 | 0.96 | 0.62 |
| **MMA% at Weeks 0 and 12** | 2.19 | 0.91 | 0.02 | 1.50 | 0.90 | 0.10 | -0.93 | 0.64 | 0.15 |
|  |  |  |  |  |  |  |  |  |  |
| **Total blood As^c^ at Week 0** | 0.08 | 0.13 | 0.57 | -0.02 | 0.13 | 0.88 | -0.12 | 0.09 | 0.20 |
| **Total blood As^c^ at Week 12** | 0.28 | 0.14 | 0.06 | 0.06 | 0.14 | 0.66 | -0.08 | 0.10 | 0.44 |
| **Total blood As^c^ at Weeks 0 and 12** | 0.15 | 0.10 | 0.11 | 0.01 | 0.10 | 0.88 | -0.10 | 0.07 | 0.14 |

^a^ Linear models adjusting for age, sex and genotyping batch were used for analyses of Week 0 and Week 12 data. Linear mixed models adjusting for age, sex and genotyping batch, random effect of week/timepoint were used for combined analysis of Week 0 and Week 12 blood arsenic data. Models for rs11191527 were also adjusted for rs9527, and vice versa.

^b^The MAF for rs61735836 (A) is 8%, the MAF for rs9527 (T) is 7% and the MAF for rs11191527 (T) is 15%.

^c^ Total blood As is the sum of blood AsIII, AsV, MMA and DMA measurements.
